# Transcriptional and functional motifs defining renal function revealed by single-nucleus RNA sequencing

**DOI:** 10.1073/pnas.2203179119

**Published:** 2022-06-13

**Authors:** Jun Xu, Yifang Liu, Hongjie Li, Alexander J. Tarashansky, Colin H. Kalicki, Ruei-Jiun Hung, Yanhui Hu, Aram Comjean, Sai Saroja Kolluru, Bo Wang, Stephen R. Quake, Liqun Luo, Andrew P. McMahon, Julian A. T. Dow, Norbert Perrimon

**Affiliations:** ^a^Department of Genetics, Blavatnik Institute, Harvard Medical School, Harvard University, Boston, MA 02115;; ^b^Department of Biology, Stanford University, Stanford, CA 94305;; ^c^HHMI, Stanford University, Stanford, CA 94305;; ^d^Department of Bioengineering, Stanford University, Stanford, CA 94305;; ^e^Chan Zuckerberg Biohub, San Francisco, CA 94158;; ^f^Department of Developmental Biology, Stanford University School of Medicine, Stanford, CA 94305;; ^g^Department of Stem Cell Biology and Regenerative Medicine, Eli and Edythe Broad Center for Regenerative Medicine and Stem Cell Research, Keck School of Medicine of the University of Southern California, Los Angeles, CA 90089;; ^h^Institute of Molecular, Cell & Systems Biology, College of Medical, Veterinary and Life Sciences, University of Glasgow, Glasgow G12 8QQ, United Kingdom;; ^i^HHMI, Harvard University, Boston, MA 02115

**Keywords:** snRNA-seq, Malpighian tubules, nephrocytes, cross-species, kidney disease

## Abstract

Recent advances in single-cell sequencing provide a unique opportunity to gain novel insights into the diversity, lineage, and functions of cell types constituting a tissue/organ. Here, we performed a single-nucleus study of the adult *Drosophila* renal system, consisting of Malpighian tubules and nephrocytes, which shares similarities with the mammalian kidney. We identified 11 distinct clusters representing renal stem cells, stellate cells, regionally specific principal cells, garland nephrocyte cells, and pericardial nephrocytes. Characterization of the transcription factors specific to each cluster identified *fruitless* (*fru*) as playing a role in stem cell regeneration and *Hepatocyte nuclear factor 4* (*Hnf4*) in regulating glycogen and triglyceride metabolism. In addition, we identified a number of genes, including *Rho guanine nucleotide exchange factor at 64C* (*RhoGEF64c*), *Frequenin 2* (*Frq2*), *Prip*, and *CG1093* that are involved in regulating the unusual star shape of stellate cells. Importantly, the single-nucleus dataset allows visualization of the expression at the organ level of genes involved in ion transport and junctional permeability, providing a systems-level view of the organization and physiological roles of the tubules. Finally, a cross-species analysis allowed us to match the fly kidney cell types to mouse kidney cell types and planarian protonephridia, knowledge that will help the generation of kidney disease models. Altogether, our study provides a comprehensive resource for studying the fly kidney.

## Significance

We performed a single-nucleus RNA sequencing study of the adult fly kidney, identifying 11 distinct clusters that we validated by gene markers. We characterized the roles of transcription factors involved in stem cell regeneration and metabolism, as well as genes that regulate the unusual cell shape of stellate cells. The dataset also provides a systems-level view of the organization and physiological roles of the tubules. Finally, we performed a cross-species analysis that allowed a comparison of the fly kidney cell types with mouse kidney cell types, as well as planarian protonephridia. This study provides a comprehensive resource for studying the insect kidney.

The functions of excretory systems are to remove toxins from the body and maintain homeostatic balance. For example, mammalian kidneys play important roles in several physiological processes, including water fluid homeostasis, removing metabolic waste products, controlling blood pressure, regulating blood cell composition, and bone mineralization (1). Although the excretory systems of various animals have differences, they typically have in common two activities: filtration and tubular secretion/reabsorption (2). In mammals, the mature kidney consists of two connected parts: a nephron, derived from the metanephric mesoderm, and a collecting tubule derived from the ureteric bud (1, 3).

The *Drosophila* renal system is composed of separated filtration nephrocytes and Malpighian (renal) tubules (4). Nephrocytes, which are first detectable at the end of embryogenesis and maintained into the adult stage, are composed of two distinct cell populations: 25 garland cell nephrocytes and 120 pericardial nephrocytes. Garland cell nephrocytes form a ring around the junction between the proventriculus and esophagus, whereas pericardial nephrocytes are located on both sides of the heart tube (5). These two types of nephrocytes, although derived from different cell lineages, share morphological, functional, and molecular features with podocytes, which form the glomerular filter in vertebrates and possess a protein sequestration activity reminiscent of the mammalian proximal tubule (5). The Malpighian tubules, considered to be analogous to the renal tubular system, develop from the ectodermal hindgut primordium and visceral mesoderm, and consist of two pairs of epithelial tubes that empty into the hindgut at its junction with the posterior midgut (6).

*Drosophila* Malpighian tubules and nephrocytes have been used to model human kidney diseases. Previously, a screen for genes involved in renal function identified over 70 genes required for nephrocyte function (7). In addition, 30 causative genes involved in steroid-resistant nephrotic syndrome have been analyzed in fly nephrocytes. Among them, *Cubilin* (*Cubn*) was found to be required for nephrocyte endocytosis (8). Furthermore, the coenzyme Q10 (CoQ10) biosynthesis gene *Coq2*, involved in regulating the morphology of the slit diaphragm-like structure and reactive oxygen species formation, contributes to a pathomechanism of COQ2-nephropathy (8). In addition to modeling numerous human renal conditions, such as chronic kidney disease and kidney stones, the Malpighian tubule is also an excellent model for studying the neuroendocrine control of renal function and rapid fluid transport (9).

Single-nucleus (snRNA-seq) and single-cell (scRNA-seq) RNA sequencing provide an opportunity to understand and revisit the cellular makeup of many organ systems, including the kidney. The mammalian kidney is composed of cell types with unique functions. Podocytes regulate the passage of proteins. Principal cells and intercalated cells balance systemic water, pH, and salt levels in the collecting duct (10–12). Two detailed scRNA-seq studies defined the whole landscape of the mouse and human kidney, with 32 distinct clusters of ontology (13, 14). Finally, scRNA-seq data can be used for the analysis of the pseudotemporal ordering of cells, which can provide information about the developmental trajectories of cellular lineages.

Here, we characterized the organization and physiological functions of the adult fly kidney at the single-cell level. Specifically, we identified 11 distinct clusters, including renal stem cells, stellate cells, principal cells, garland nephrocytes cells, and pericardial nephrocytes, and provide gene-expression–level data at single-cell resolution. Our study provides a comprehensive resource to study the fly kidney. For example, we identified the function of transcription factors (TFs) and genes that control cell shape. In addition, we performed a cross-species analysis between the fly kidney, planarian protonephridia, and mouse kidney, allowing us to map kidney cell types across species.

## Results

### snRNA-seq Identifies 11 Distinct Clusters in the Adult *Drosophila* Kidney.

**Fig. 1.**
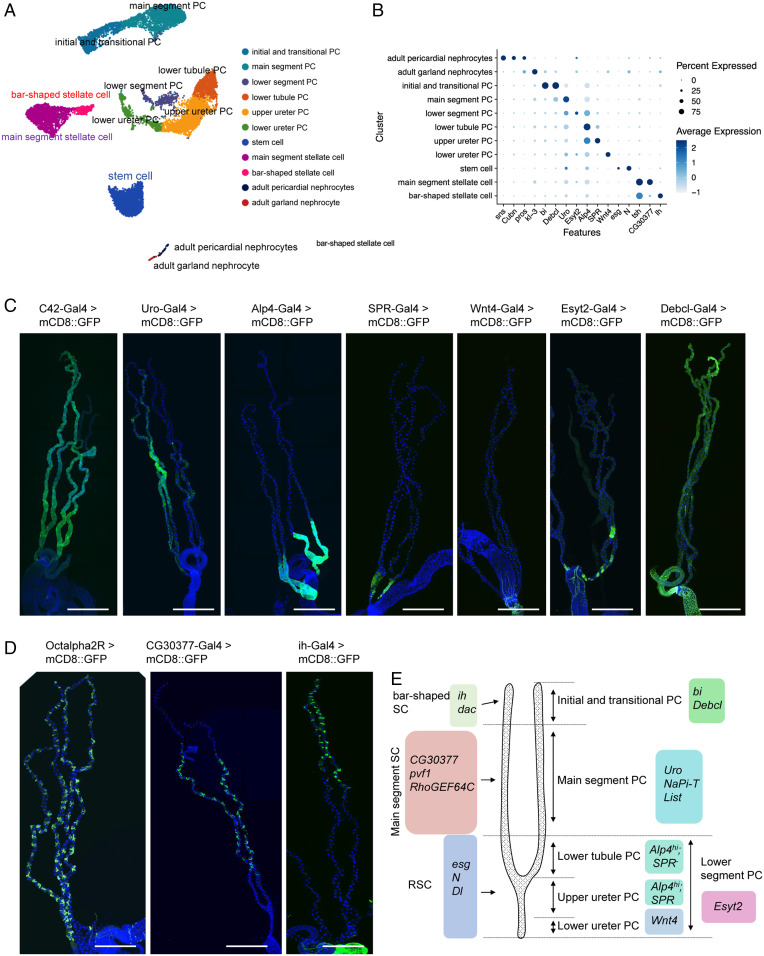
snRNA-seq analysis and markers identification of the fly adult kidney. (*A*) A single UMAP of the “fly kidney” contains 11 distinct cell clusters that were annotated on the UMAP. Note, however, that nephrocytes and tubules are not physically associated in vivo. (*B*) Expression levels and percentage of cells expressing the marker genes in each cluster are shown as a dot plot. (*C* and *D*) GFP expression under the control of Gal4 lines specific for each of the principal cell (PC) and stellate cell (SC) clusters. Note that the Gal4 expression patterns of *SPR*, *Wnt4*, *Esyt2*, *Debcl*, *Octalpha2R*, *CG30377*, and *ih* have not been previously reported. (Scale bars, 500 μm.) (*E*) Malpighian tubule cell types are identified based on differentially expressed marker genes.

The fly kidney consists of Malpighian tubules and nephrocytes that are located in different regions of the body. As part of the Fly Cell Atlas project, we dissected male and female Malpighian tubules (15) and annotated the cell types. In addition, as nephrocytes were not included in the Fly Cell Atlas, we performed snRNA-seq of both garland nephrocyte cells and pericardial nephrocytes (see details on sample and sequencing information in Dataset S1). We successfully recovered 12,166 nuclei in the tubules, identified a garland cell nephrocyte cluster with 41 nuclei, and a pericardial nephrocyte cluster with 93 nuclei. In addition, 11 distinct clusters representing renal stem cells, stellate cells, regionally specific principal cells, and nephrocyte cells were identified (Fig. 1 *A* and *B*; marker genes listed in Dataset S2). Note that the tubule snRNA-seq data were independently annotated at Harvard and by the Fly Cell Atlas group with highly concordant results (*SI Appendix*, Fig. S2). In addition, we validated new markers, identified as cluster-specific, by driving fluorescent reporters with the appropriate GAL4 lines (Fig. 1 *C* and *D* and Dataset S3).

The Malpighian tubule stem cell cluster is defined by the expression of *escargot* (*esg*), *Notch* (*N*), and *Delta* (*Dl*) genes (16). In addition, we identified six principal cells clusters (initial and transitional principal cells, main segment principal cells, lower tubule principal cells, upper ureter principal cells, lower ureter principal cells, and lower segment principal cells). Initial and transitional principal cells express *bifid* (*bi*) and *Death executioner Bcl-2* (*Debcl*) (Fig. 1*C*). The main segment principal cells express *urate oxidase* (*Uro*) (16) (Fig. 1*C*). Markers for lower tubule principal cells, upper ureter principal cells, lower ureter principal cells, and lower segment principal cells are: *SPR*, *Alp4*^hi^; *SPR*, *Alp4*^hi^; *Wnt4*, *Alp4*^hi^; and *Esyt2*, respectively (Fig. 1 *B* and *C*). *Alkaline phosphatase 4* (*Alp4*) has previously been reported as a marker of all principal cells in the lower segment (17) (Fig. 1*C*). In addition, we identified a small cell cluster expressing *Extended synaptotagmin-like protein 2* (*Esyt2*) that may represent a previously unrecognized lower segment cell cluster. *Esyt2* is expressed only in some cells in the lower segment, which correspond to an independent cluster on the UMAP. Gene ontology terms of *Esyt2*-expressing cells include “cell morphogenesis involved in differentiation,” “cell morphogenesis involved in neuronal differentiation,” and “actin-filament based process” (detailed information in *SI Appendix*, *Supplementary Text* and Fig. S2).

In addition, we identified two stellate cell clusters (bar-shaped stellate cells located in the anterior tubules and main segment stellate cells) (18) that both express *teashirt* (*tsh*), *kinin receptor* (*lkr*), and *Secretory chloride channel* (*SecCl*) (19–21). We also characterized a number of specific markers: *I_h_ channel* (*ih*) for bar-shaped stellate cells (Fig. 1*D*), *CG30377* for main segment stellate cells, and *α2-adrenergic-like octopamine receptor* (*Octα2R*) for all stellate cells (Fig. 1*D*).

The anterior and posterior tubules differ in morphology (22), gene expression (18, 23), and function (24). Specifically, anterior tubules have more prominent initial and transitional segments and play a major role in calcium excretion. Although anterior and posterior tubules were not separated during this dissection, they are nonetheless separable in our data by reference to the published bulk transcriptomes of anterior and posterior tubules (23). Both principal and stellate cells of the anterior tubule express the *Dorsocross* genes (*Doc1-3*), allowing the identification of bar-shaped cells, which are related to—but distinct from—the stellate cell cluster. Similarly, the initial/transitional cell cluster, which is enriched in *bi* and *Debcl*, is distinguishable from the main segment principal cells (Fig. 1). Within the main segment and lower tubule, *Dorsocross*-marked cells intersperse randomly with others, suggesting that the two tubule pairs are indistinguishable in their main and lower segment transcriptomes, but have a unique initial/transitional transcriptome.

Nephrocyte clusters are defined by the expression of *sticks and stones* (*sns*), *Cubn*,* Hand*, and *prospero* (*pros*) (25–27). *Sns* encodes a core component of the slit diaphragm-like structure (22) and *Cubn* encodes a receptor for protein reabsorption (26); both are critical for the function of nephrocytes. These two genes have lower expression in garland nephrocytes cells compared to pericardial nephrocytes. Details on the marker genes are listed in Dataset S3. *Wnt4*, *SPR*, *Esyt2*, *Debcl*, *ih*, *CG30377*, and *Octα2R* are marker genes that we validated in this study. Finally, in order to make the dataset accessible to users, we developed a visualization web portal (https://www.flyrnai.org/scRNA/kidney/) that allows users to query the expression of any gene of interest in different cell types.

### Cell-Type–Specific Expression of TFs and Regulatory Landscape.

**Fig. 2.**
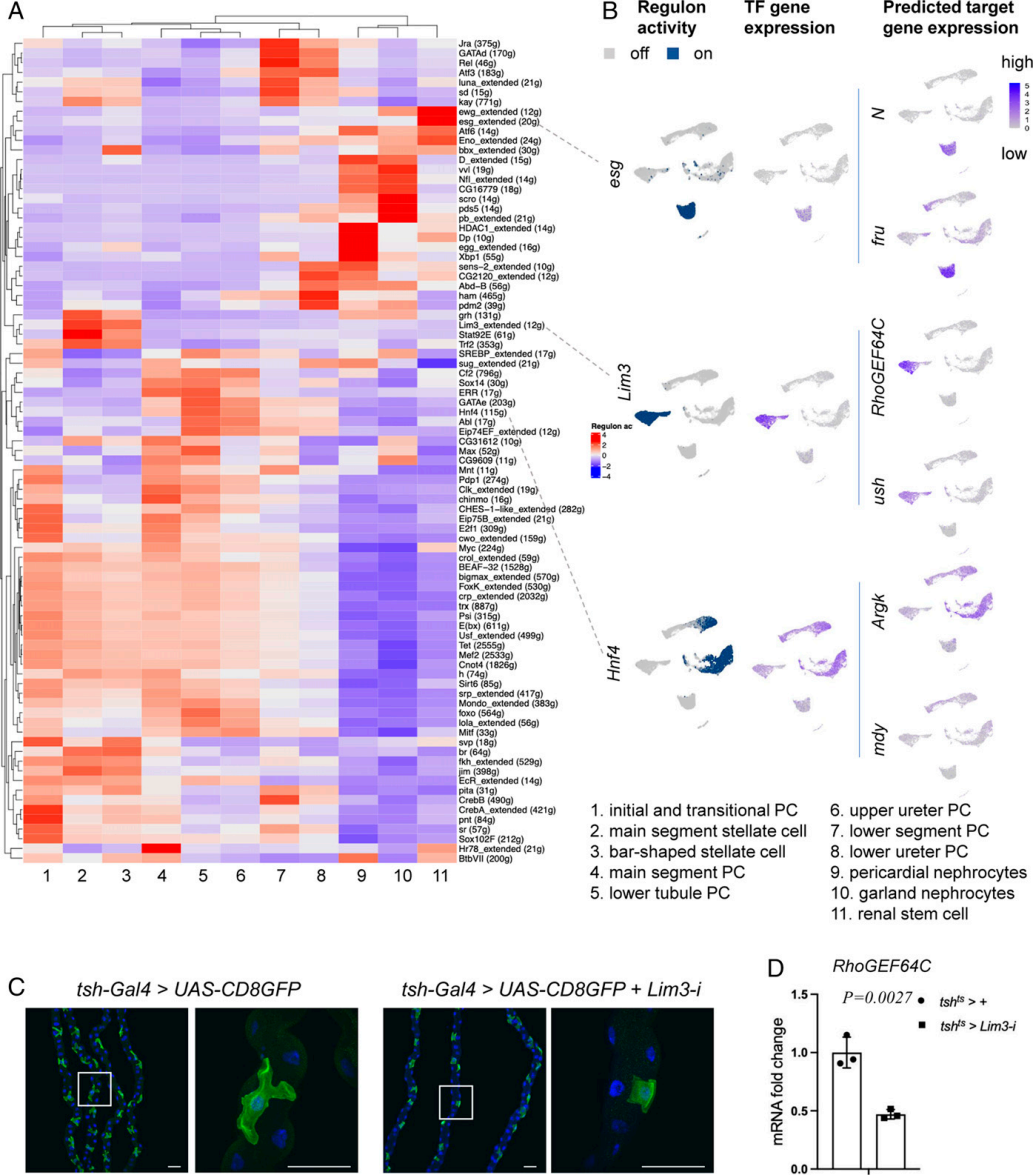
Cell-type–specific gene regulatory landscape of the fly kidney. (*A*) SCENIC results of the fly kidney. The heatmap shows the gene-expression level in each cluster. Low regulon activity is shown with blue color and high regulon activity is shown in red. See *SI Appendix*, Fig. S8 for an enlarged version of the heat map with gene names. (*B*) UMAP depiction of regulon activity (“on-blue,” “off-gray”) and TF gene expression (blue scale) of renal stem cells (*esg*), stellate cells (*Lim3*), and principal cells (*Hnf4*). Examples of target gene expression of the *esg* regulon (*N* and *fru*), *Lim3* regulon (*RhoGEF64C* and *u-shaped* [*ush*]), and *Hnf4* regulon (*Argk* and *mdy*) are shown in blue. (*C*) Stellate cell shape phenotype associated with RNAi knockdown of *Lim3*. DAPI (blue) staining for nuclei. (Scale bars, 50 μm.) (*D*) qPCR results show the *RhoGEF64C* mRNA fold change of *tsh^ts^ > +* and *tsh^ts^ > Lim3-i* in the Malpighian tubules for 8 d.

To investigate the TFs that may contribute to kidney differentiation and function, we identified 44 cell-type–specific TFs by setting up the parameter cutoff based on gene-expression levels (fold-change > 3) and adjusting the *P* value (<0.05) (*SI Appendix*, Fig. S3*A*). We also applied SCENIC (single-cell regulatory network inference and clustering) to reveal TF-centered gene coexpression networks (28) for the simultaneous reconstruction of gene-regulatory networks and identification of cell states (Fig. 2 *A* and *B*). By inferring a gene-correlation network followed by motif-based filtration, SCENIC keeps only potential direct targets of each TF as modules (regulons). Among the TFs, *esg*, *klumpfuss* (*klu*), and *Sox100B* are specifically expressed in renal stem cells, and SCENIC could infer multiple downstream target genes. For example, among the *esg* target genes are *fruitless* (*fru*), *N*, *Dl*, and *klu* (Fig. 2*B* and Dataset S4). *fru* is expressed in renal stem cells of both sexes (*SI Appendix*, Fig. S3*B*) and knockdown of *fru* using EGT (*esg-GAL4*, *UAS-GFP*, *tub-GAL80^TS^*) induced renal stem cell proliferation, suggesting that it plays a role in renal stem cell proliferation and maintenance (*SI Appendix*, Fig. S3*C*). Another TF expressed in renal stem cells is *Sba* (*six-banded*). Interestingly, some of the TFs expressed in renal stem cells are also expressed in other types of stem cells. For example, *esg*, *klu*, and *Sox100B* are essential for intestinal stem cell proliferation and maintenance (29). In addition, *fru* is expressed in the male gonad stem cell niche and plays important roles in the development and maintenance of germline stem cells (30). *tsh*, *tiptop* (*tio*), and *Lim3* are expressed in both main segment and bar-shaped stellate cells (*SI Appendix*, Figs. S2*B* and S3*A*). *tsh* and *tio* are paralogous genes that control stellate cells shape and the expression of genes required for terminal physiological differentiation (21, 31). Interestingly, human *TSHZ* genes (orthologs of *tsh*) are causal kidney disease loci, including ureteral smooth muscle differentiation and congenital pelvi–ureteric junction obstruction (32, 33). In addition, we found that *dachshund* (*dac*), *Doc1*, and *Doc2*, previously reported to be expressed in initial and transitional principal cells and involved in tissue morphogenesis (34–36), are also highly expressed in bar-shaped stellate cells (*SI Appendix*, Fig. S3 *A* and *B*), suggesting that they may also have a role in their morphogenesis.

One interesting TF gene expressed in several subset cell clusters of principal cells is *Hepatocyte nuclear factor 4* (*Hnf4*). *Hnf4* human orthologs are *Hnf4γ* and *Hnf4α*, major regulators of renal proximal tubule development in the mouse (37). Purine metabolites—including inosine, adenine, xanthine, hypoxanthine, and uric acid—are associated with increased diabetes risk and diabetic nephropathy, and are increased in *Hnf4* mutant flies (38). Potential direct targets of Hnf4 include *Arginine kinase* (*Argk*) and *midway* (*mdy*) (Fig. 2*B*), with *mdy* acting as a repressor of *Hnf4* and HNF4 controlling lipid metabolism in *Drosophila* nephrocytes (39). We found that *Hnf4* is expressed only in principal cells, but not in stellate and renal stem cells (Fig. 2*C*). Interestingly, knockdown of *Hnf4* in the principal cells increased whole-body level of glycogen and TAG, suggesting that it regulates glycogen and TAG metabolism in Malpighian tubules (*SI Appendix*, Fig. S3*D*). Altogether, our analysis provides a list of TFs that control intestinal stem cells maintenance, stellate cells morphology, and kidney physiological function. Additional information on these TFs can be found in Dataset S5.

### Control of Stellate Cell Shape.

**Fig. 3.**
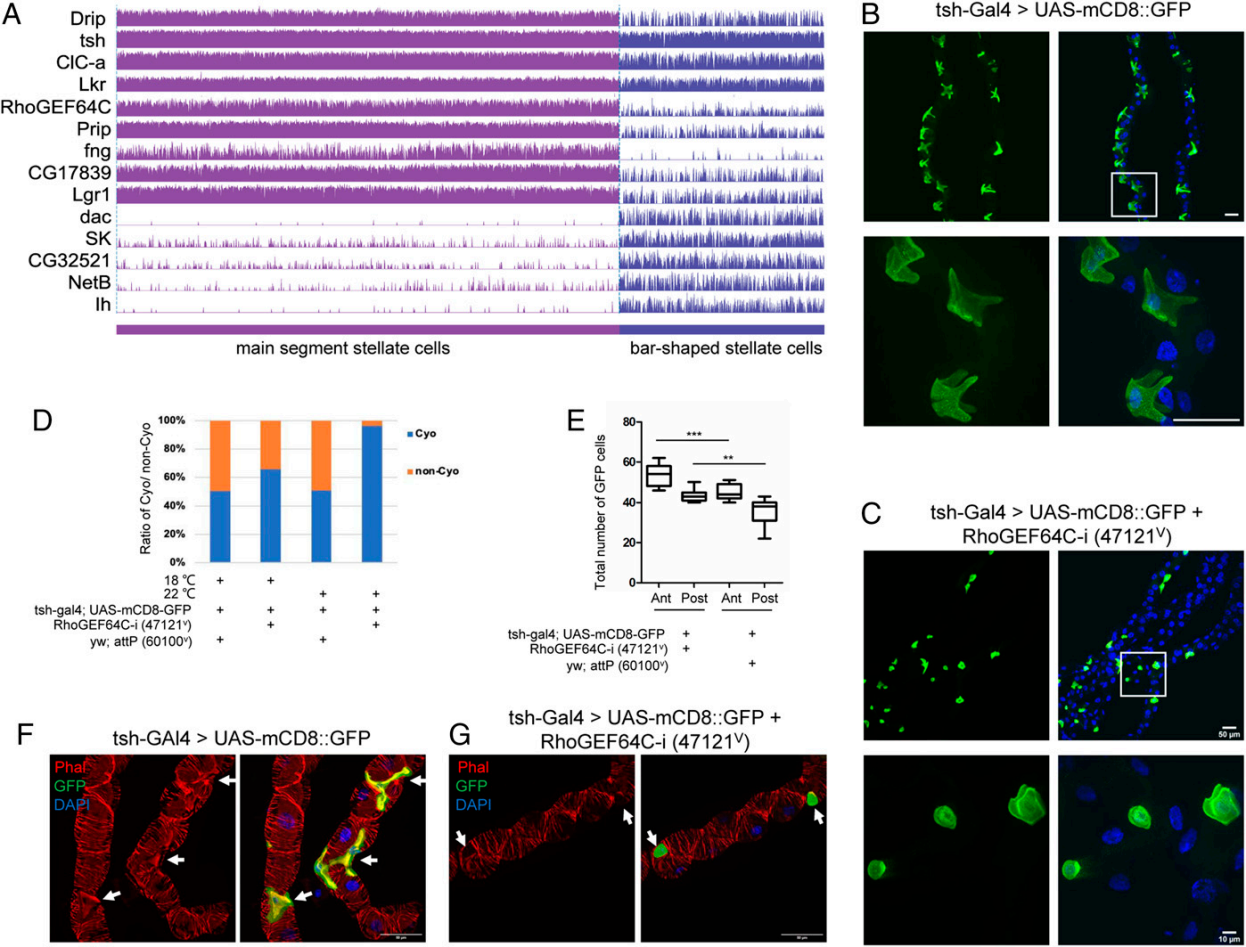
*RhoGEF64c* maintains stellate cell shape. (*A*) Gene-expression levels of selected markers specifically expressed in stellate cells. (*B*) Cell shape visualized using tsh-Gal4 driving mCD8-GFP. DAPI (blue) is used to stain nuclei. White box indicates the zoom-in region. (Scale bars, 50 μm.) (*C*) Knockdown using VDRC line 47121^v^ of *RhoGEF64c* affects stellate cell shapes. (Scale bars, 50 μm and 10 μm.) (*D*) Survival of *RhoGEF64c* knockdown (47121^v^) and control (60100^v^) animals. Statistics of CyO or non-CyO flies from the adult progenies *RhoGEF64c* knockdown and control raised at 18 °C and 22 °C. (*E*) Statistics of cell number of stellate cells (GFP^+^) of in *RhoGEF64c* knockdown and control flies. “Ant” means anterior Malpighian tubules and “post” means posterior Malpighian tubules. Data are presented as means ± SEM. **P < 0.01, ***P < 0.001. (*F* and *G*) Knockdown of *RhoGEF64c* results in loss of cytoarchitectural organization. Cell cytoarchitecture is visualized by Phalloidin (Phal; F-actin) staining. Arrows indicate SCs. (Scale bars, 50 μm.)

Stellate cells, which control channel-mediated Cl^−^ and water flux, have a cuboidal shape in third-instar larvae. Subsequently, during the pharate adult stage, they adopt a star shape in the main segment and a bar shape in the initial segment (40–43). Previous studies have shown that disruption of stellate cells affects fly survival. For example, conditional down-regulation of *Rab11* in stellate cells results in lethality at the pharate adult stage, and knockdown of *Snakeskin* (*Ssk*) in stellate cells results in loss of fluid integrity and a significant reduction in viability (43, 44). Furthermore, ablation of stellate cells causes lethality, confirming the essential role of this cell type (45). We identified two subclusters of stellate cells: bar-shaped stellate cells and main segment stellate cells (see Fig. 3*A*). Bar-shaped cells were originally described as a morphologically distinguishable subset of cells found only in initial segments of anterior tubules (18). These cells express *tsh*, as revealed by the c710 and c724 GAL4 lines that are both inserted downstream of *tsh*. Since then, we and others have shown that stellate cells also express the kinin receptor, the chloride channels Clc-a and SecCl, and the water channels Drip and Prip (41). Interestingly, bar-shaped cells do not express any of the key genes for transport in our dataset, even though they share a common developmental pathway, suggesting that they are likely not able to transport fluid. This is consistent with physiological studies that attempted to map transport along the length of the tubule, showing that initial segments do not transport fluid (46). Thus, our study indicates that, although stellate cells and bar-shaped cells share a common developmental pathway, they are both functionally and structurally separable.

To study the physiological function of stellate cells, we knocked down the top marker genes defining this cluster using *tsh-Gal4*, focusing on their shape and fly viability (*SI Appendix*, Fig. S4*A*). Among the 18 genes analyzed, 13 were associated with reduced viability, 4 affected main segment stellate cells shape, and 2 reduced main segment stellate cell number (*SI Appendix*, Fig. S4 *B*–*F*). Among these genes, the top-ranking marker gene, *Rho guanine nucleotide exchange factor at 64C* (*RhoGEF64C*) (Fig. 3*A*), encodes an exchange factor for Rho GTPases. Knocking down *RhoGEF64c* affects cell shape of the main segment stellate cells (Fig. 3 *B* and *C*), viability (Fig. 3*D*), and their number (Fig. 3*E*). In humans, Rho-GTPases regulate the formation and maintenance of long cellular extensions/foot processes and their dysfunctions are associated with nephrotic syndrome (47). Furthermore, following podocyte injury, Rho-GTPases orchestrate the rearrangement of the actin cytoskeleton (47). Interestingly, knockdown of *RhoGEF64c* results in loss of cytoarchitectural organization in main segment stellate cells (Fig. 3 *F* and *G*) but did not affect septate junctions (*SI Appendix*, Fig. S5 *A* and *B*). This contrasts with knockdown of *Ssk,* which caused loss of both cytoarchitectural organization and septate junctions (44). Finally, knockdown of other top marker genes—namely *Prip*, *Frq2*, and *CG10939*—did not affect the septate junctions (*SI Appendix*, Fig. S5 *C*–*E*).

Interestingly, SCENIC results reveal that Lim3 is a highly enriched TF in stellate cells (Fig. 2*B*) and that *RhoGEF64C* is one of its predicted targets (*SI Appendix*, Fig. S3*B*). Consistent with the role of *RhoGEF64C* in controlling stellate cell morphology, knockdown of *Lim3* is associated with controlling stellate cell morphology and the mRNA level of *RhoGEF64C* (Fig. 2 *C* and *D*). Altogether, our results suggest that the star shape is essential for normal physiological function of stellate cells in adults.

### Reconciling Physiology with Clusters.

**Fig. 4.**
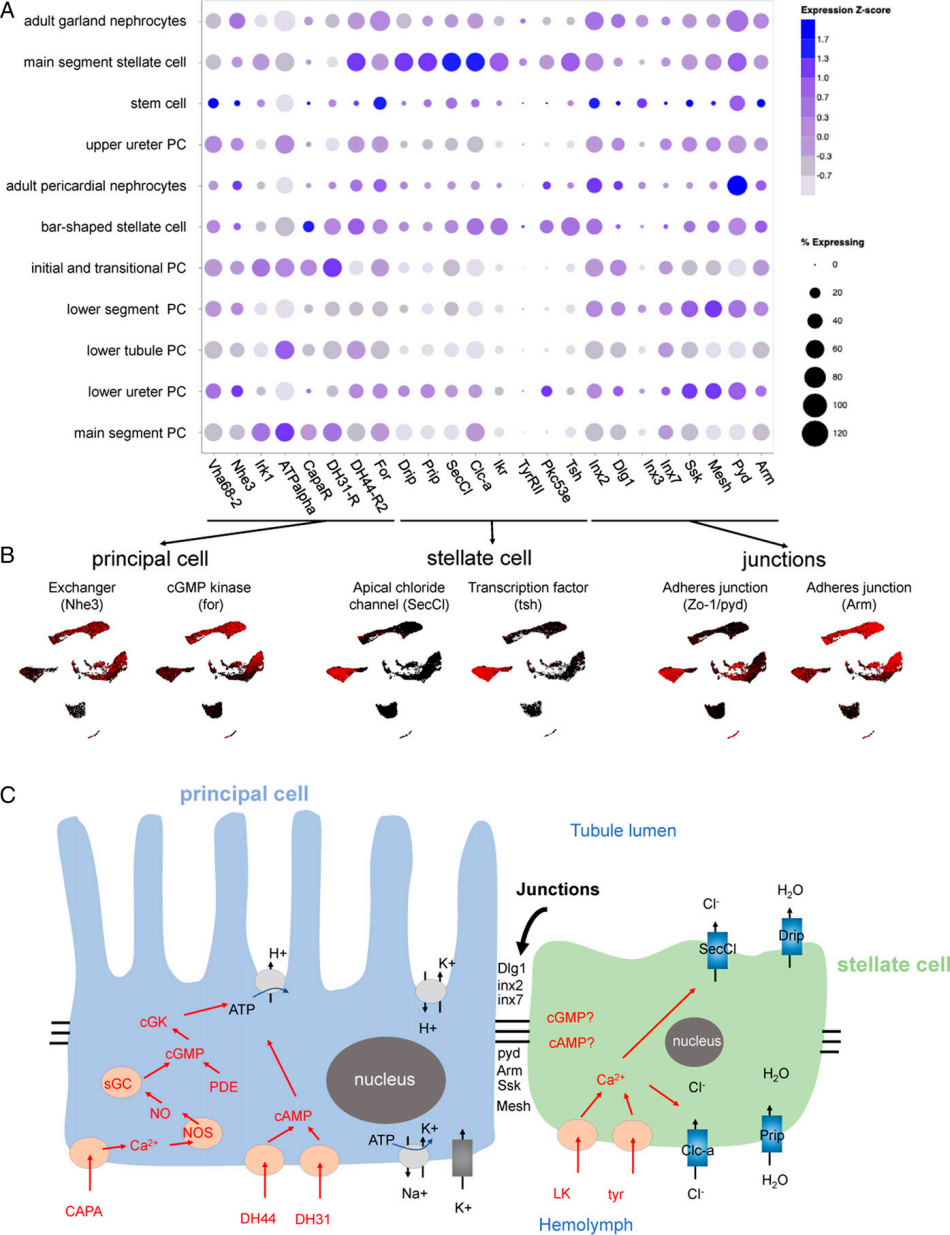
Mapping function to cell types and regions in the tubule. (*A* and *B*) t-Distributed stochastic neighbor embedding (tSNE) and UMAP distribution of genes involved in principal cell, stellate cell, and junctions. (*C*) Overview of the two-cell model of insect tubule fluid secretion and its control. Adapted from ref. (84).

The Malpighian tubule generates a primary urine not by paracellular filtration but by potent active cation transport. In *Drosophila*, this is coupled to channel-mediated anion flux to balance charge and water channels to allow rapid flux of osmotically obliged water (9). The tubules can transport their own volume of water every 6 s, making them the fastest-secreting epithelium known (48). In contrast with the vertebrate nephron, the paracellular route in Malpighian tubules depends on septate junctions and solutes are excreted by a wide range of transporter genes (49–52). Many of the genes underlying these processes have been identified and some of them were specifically expressed in some cell types. However, the single-nucleus datasets allow us to address various questions at a larger scale. In particular, are genes ascribed to particular processes expressed in the same cell types or regions? What new insights can be gained as to regional specialization, and can we predict functions of previously uncharacterized genes based on their expression patterns (Fig. 4)?

Gene expression of principal cells in the UMAP broadly follows expectation (Fig. 4 *A* and *B*). First, the plasma membrane V-ATPase subunit genes all show elevated expression in principal cells along the whole length of the tubule, not just in the main segment (Fig. 4*A* and *SI Appendix*, Fig. S6*A*). Second, a candidate apical exchanger, *Na^+^/H^+^ hydrogen exchanger 3* (*Nhe3*), has the same expression pattern as V-ATPase subunit genes, which is also similar to Na^+^, K^+^ ATPase that stabilizes cellular cation levels (53) (Fig. 4 *A* and *B* and *SI Appendix*, Fig. S6*B*). This implies that the basic transport machinery is an inherent property of the whole length of the tubule, not just the secretory region. In contrast, the inward rectifier K^+^ channel family genes, all of which are strongly expressed in the tubule, show distinct patterns. *Inwardly rectifying potassium channel* (*Irk) 1* marks principal cells of only the secretory main segment of the tubule (Fig. 4*A*), *irk2* is expressed in the main segment and lower tubule, and *irk3* is generally expressed in principal cells (*SI Appendix*, Fig. S6*C*). Furthermore, receptors for the three major neuropeptides (Capa, DH31, and DH44) are found in principal cells of the main segment. These hormones are believed to act on principal cells to stimulate secretion. However, their expression patterns are slightly different (Fig. 4*A*). Both the *Capa receptor* (*CapaR*) and *Diuretic hormone 31 Receptor* (*DH31-R*) are expressed in the initial, transitional, and main segment. However, *DH44-R2* is present in the main segment, lower tubule, and surprisingly, in stellate cells.

Stellate cells are thought to provide a transcellular shunt for anions and water, and accordingly, the two chloride channels, *Chloride channel-a* (*Clc-a*) (54) and *Secretory chloride channel* (*SecCl*) (21), as well as the two true aquaporins *Drip* and *Prip* (41), show strong stellate cell-enriched expression (Fig. 4 *A* and *B*). Kinin and tyramine act on the renal tubule stellate cell to activate chloride shunt conductance (55) and both their receptors show strong localization to stellate cells, together with their downstream effector, protein kinase C, and the master transcription factor, *tsh*, which specifies stellate cell fate (21) (Fig. 4*A*). However, in our dataset, none of these genes show strong expression in bar-shaped cells, suggesting that although they are developmentally linked to stellate cells, bar-shaped cells are not able to either receive diuretic signals or respond to them.

Junctional permeability is critical in epithelia. As the principal cell and stellate cell lineages have distinct origins (ectodermal and mesodermal, respectively) (45), they might not necessarily form heterotypic junctions. In fact, both cell types express the septate (occluding) junction-associated genes *discs large 1* (*dlg1*), *ssk* (44), and *Mesh* (56) throughout the length of the tubule (Fig. 4*A*), suggesting that both principal cell–principal cell and principal cell–stellate cell junctions are equally tight. Interestingly, although both cell types also contribute adherens junction components, their marker genes are different, with *polychaetoid* (*ZO-1/pyd*) being highly expressed in stellate cells and *armadillo* (*arm*) in principal cells (Fig. 4 *A* and *B*). Of the gap junction (innexin) genes, three are strongly expressed in the tubule (57); *inx2* and *inx7* are expressed in principal cells but not stellate cells, and *inx3* transcripts are enriched in stem cells. Thus, principal cells have the ability to communicate and synchronize activities, but stellate cells are likely to be functionally independent. There is experimental evidence to support this idea in *Drosophila*; stimulation of principal cells with Capa elevates intracellular calcium in principal cells but not stellate cells (58), whereas the opposite holds for Kinin signaling (21). *CapaR* and *Kinin receptor* (aka *Leucokinin receptor -* *Lkr*) are expressed in principal cells and stellate cells, respectively (Fig. 4*A*). The Capa and Kinin pathways thus act independently on two cell types without detectable cross-talk (59). Functionally, stimulation of principal cells by Capa does not activate chloride shunt conductance, a hallmark of stellate cell activation by Kinin, indicating that calcium signals do not pass between the cell types (60). Note that although *Drosophila* provides a valuable reference, the phylogenetic breadth of insects is not to be underestimated, and this model may not apply universally; for example, the distal iliac plexus of the Lepidopteran *Trichoplusia ni* tubule may behave differently with respect to cell–cell coupling and reversibility of fluxes (61–63).

The tubules show strongly enriched expression of genes encoding organic solute transporters (49), including the ABC-transporters that underly eye color (*white* [*w*], *scarlet* [*st*], *brown* [*bw*]), and these are all confined to main segment principal cells (*SI Appendix*, Fig. S6*D*). Tubules are also excellent models for renal diseases (9, 64) and readily develop oxalate kidney stones. Knockdown of the oxalate transporter *Prestin* increases kidney stone prevalence, presumably by preventing reuptake of secreted oxalate (65, 66); *Prestin* is expressed in principal cells of the reabsorptive (67) lower tubule (*SI Appendix*, Fig. S6*D*). Similarly, transporter genes that have been implicated in the excretion of xenobiotics (53) are expressed only in principal cells (*SI Appendix*, Fig. S6*E*), confirming the role of these cells in general-purpose solute transport.

As well as transport, tubules play a liver-like role in detoxification, and show expression of genes known to detoxify insecticides (e.g., *Cytochrome P450 6g1* [*Cyp6g1*] and *Cyp12d1*) (68–70), and the master transcriptional regulator *Hormone receptor-like in 96* (*Hr96*) (71); all of these genes show close coexpression in principal cells (*SI Appendix*, Fig. S6*F*). Several transcription factors allow the clusters imputed here to be resolved. For example, *tsh* and *tio* are stellate cell-specific, *N* marks stem cells, and *Dac*, *Doc1*, Homothorax (*Hth*), and *cut* (*ct*) provide graded resolution of principal cell domains (*SI Appendix*, Fig. S6 *G*–*I*).

### Cross-Species and Human Kidney Disease Analysis.

**Fig. 5.**
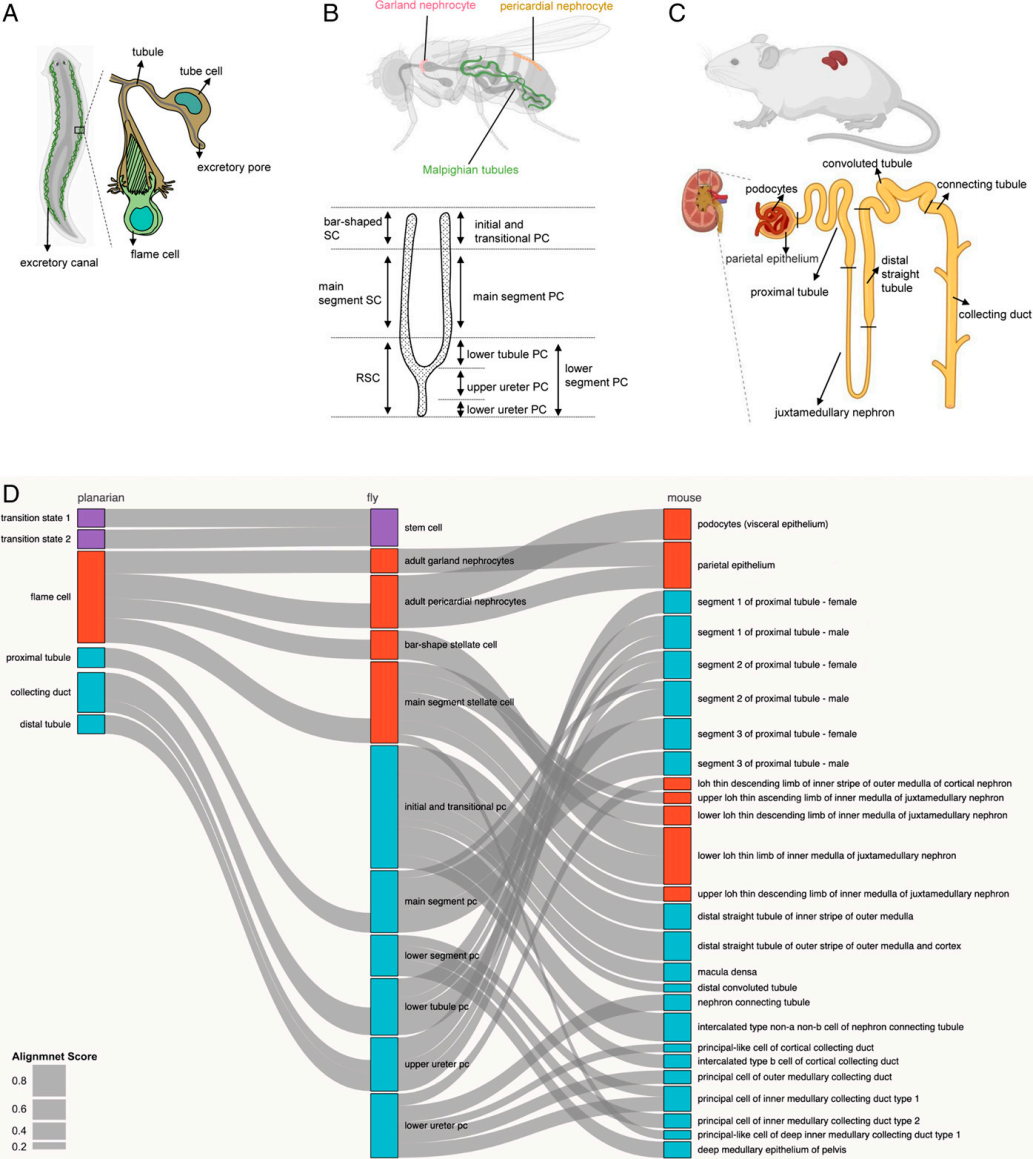
Cross-species analysis of fly, planarian, and mouse kidneys using SAMap. (*A*–*C*) The cartoons show the structure of the kidneys of three species. The planaria excretory canal contains tubule cells and flame cells. The fly kidney contains Malpighian tubules and two types of nephrocytes. The mouse kidney unit contains collecting duct, connecting tubule, convoluted tubule, distal straight tubule, juxtamedullary nephron, proximal tubule, podocytes, and parietal epithelium. (*D*) Sankey plot summarizing the cell-type mappings. Edges with alignment scores < 0.1 were omitted. Nonstem cell types are arranged along the proximal-distal axis. Magenta: stem cell types; red: ultrafiltration cell types; blue: resorption cell types.

Considering that the function of all animal excretory systems is to remove toxins from the body and maintain homeostatic balance, we next asked whether we could match fly kidney cell types to higher animal kidney cell types (mouse) and lower animal protonephridia cell types (planarian) (Fig. 5 *A*–*C*), and whether the single-cell–level data can help implicate new genes and cell types in human kidney diseases. We used the Self-Assembling Manifold mapping (SAMap) algorithm (72) to map our fly single-cell transcriptomes with scRNA-seq data from mouse (13) and planaria (73). This method depends on two modules. First is a gene–gene bipartite graph with cross-species edges connecting homologous gene pairs weighted by cell-type–specific expression similarity (all gene pairs are listed in Datasets S5 and S6). Second is a gene–gene graph projecting two single-cell transcriptomic datasets into a joint manifold representation, from which each cell mutual cross-species neighbors are linked to stitch the cell atlases together for fly and mouse kidney (*SI Appendix*, Fig. S7 *A* and *B*). With this method, SAMap produced a combined manifold with a high degree of cross-species alignment (*SI Appendix*, Fig. S7*C*). After measuring the mapping strength between cell types by calculating an alignment score (as edge width, shown in Fig. 5*D*), which was defined as the average number of mutual nearest cross-species neighbors of each cell relative to the maximum possible number of neighbors, we generated a Sankey plot with 10 fly kidney cell clusters matched to 26 mouse kidney cell clusters (Fig. 5*D*). A similar analysis was performed for flies and planarians, with nine fly kidney cell clusters matched to six planarian protonephridia cell clusters (Fig. 5*D* and *SI Appendix*, Fig. S8 *A*–*C*).

The results of the fly/mouse analysis suggest that fly main segment principal cells, lower tubule principal cells, and upper ureter principal cells are similar to mouse proximal tubules (segments 1 to 3); fly bar-shaped and main segment stellate cells map to the mouse lower loop of Henle (LOH) thin limb of inner medulla of juxtamedullary nephron; fly adult pericardial nephrocytes are similar to mouse podocytes (visceral epithelium) and parietal epithelium; and fly adult garland nephrocyte cells map to mouse parietal epithelium (Fig. 5*D*). Thus, although pericardial and garland nephrocytes are frequently considered to be interchangeable, they represent different facets of the mammalian nephron. Interestingly, we found that the fly lower segment principal cells represent a discontinuous cell population located in the lower segment region and match to mouse principal cells of inner medullary collecting duct type 1/2, suggesting that the fly lower segment principal cells cluster is a Malpighian tubule cell type. Finally, to determine the specificity of our cross-species analysis, we compared mouse kidney cells (15 cell clusters) to the entire Fly Cell Atlas dataset (252 cell clusters) (15). The results showed that the mouse clusters align well with the fly Malpighian tubule clusters if the entire Fly Cell Atlas was used (*SI Appendix*, Fig. S9*A*).

The fly/planaria comparison suggests that fly stem cells are similar to planarian transition state 1 and transition state 2 tissue-specific progenitors, indicating that kidney stem cells are present in both lower animal species but not mammals. Fly main segment principal cells map to planarian proximal tubule; fly lower tubule principal cells map to the planarian collecting duct; and fly upper ureter principal cells and initial and transitional principal cells map to the planarian distal tubule. Interestingly, fly pericardial nephrocytes, garland nephrocytes, bar-shaped and main segment stellate cells map to planarian flame cells, suggesting that these cell types have conserved function for removing waste materials (Fig. 5*D*).

Next, we chose several genes from homologous gene pairs (Datasets S6 and S7) to test the robustness of the comparative analyses. Based on the SAMap, fly *PDGF- and VEGF-related factor 1* (*Pvf1*) and *tsh* are highly expressed in stellate cells. Strikingly, the corresponding mouse genes *pdgfa* and *Tshz2* are highly expressed in the lower LOH thin limb of inner medulla of juxtamedullary nephron (*SI Appendix*, Fig. S7*D*). Furthermore, fly *Cyp6g1* and *Na^+^-dependent inorganic phosphate cotransporter* (*NaPi-T*) genes are highly expressed in main segment principal cells, and their corresponding genes in the mouse, *Cyp4b1* and *Slc22a6*, are highly expressed in mouse proximal tubules (*SI Appendix*, Fig. S7*D*). *Esyt2* is a marker gene for the fly lower segment principal cells, and its orthologous gene *Esyt1* is highly expressed in principal cells of the inner medullary collecting duct type 1/2 (*SI Appendix*, Fig. S7*D*). In the fly, *sns* is highly expressed in nephrocytes, and the orthologous gene in the mouse, *Nphs1*, is highly expressed in mouse podocytes (*SI Appendix*, Fig. S7*D*). With regards to planaria, despite the lower extent of genome annotation, we identified some informative gene pairs (Dataset S7) that include the fly nephrocyte marker gene *sns*, the stellate cell marker gene *Neprilysin 2* (*Nep2*), the stem cell marker gene *esg*, and the main segment principal cell marker gene *salt*, which could be mapped to the planaria cell clusters (*SI Appendix*, Fig. S8*D*). Altogether, these results indicate that the SAMap mapping results are well supported by conserved gene-expression programs.

SCENIC results showed that there are 47 unique fly TFs that can be mapped to 94 orthologs in the mouse (*SI Appendix*, Fig. S9*B*). Comparing the role of these TFs across species can suggest novel physiological kidney functions. For example, in flies loss of *Hnf4* is associated with an increase in the level of purine metabolites, including inosine, adenine, xanthine, hypoxanthine, and uric acid (38). Studies in mice on the other hand have only reported that loss of *Hnf4α* is associated with renal proximal tubule development (37), suggesting that *Hnf4α* may also play a role in regulating kidney physiology. CellChat (74) analysis showed that 12 signaling pathways were prominent in the mouse kidney, including EGF, FGF, GAS, GRN, MIF, MIK, MK, ncWNT, NRG, PTN, SPP1, VISFATIN, and WNT. Three of them (EGF, FGF, and WNT) were also expressed in the fly renal system (*SI Appendix*, Fig. S9*C*). Previous studies in flies showed a role for FGF-signaling in the maintenance of hemolymph phosphate homeostasis through *Na^+^-dependent inorganic phosphate cotransporter* (*MFS2*)-mediated phosphate excretion by the Malpighian tubules (75), while a mouse study also showed that FGF23 regulates phosphorus homeostasis (76). We also performed a Kyoto Encyclopedia of Genes and Genomes (KEGG) metabolic pathways enrichment analysis in the mouse kidney. Among the highly/ubiquitously expressed metabolic pathways in mice, purine metabolism, glycerophospholipid metabolism, nicotinate and nicotinamide metabolism, and starch and sucrose metabolism were also observed to be highly/ubiquitously expressed in flies (*SI Appendix*, Fig. S9*D*). We also found 50 pathways that are specifically expressed in only one or two cell types in mouse. For example, five metabolic pathways comprising caffeine metabolism, nitrogen metabolism, arginine and proline metabolism, folate biosynthesis, and thiamine metabolism were predicted to be active, which is consistent with the fly result (*SI Appendix*, Fig. S9*D*). In summary, these comparative analyses provide more insights into the conserved molecular mechanisms of kidney function across species.

We also examined whether the single-nucleus data can help implicate cell clusters and gene targets in human kidney diseases, especially as a previous study in the mouse has shown that hereditary human kidney diseases characterized by the same phenotypic manifestations originate from the same cell types (77). Strikingly, single-cell distribution of human kidney diseases in the fly kidney showed that most of these genes were enriched in the orthologous cell types (*SI Appendix*, Fig. S10). In particular, the fly orthologs of 13 of 33 genes associated with monogenic inheritance of nephrotic syndrome in humans were expressed in fly nephrocytes. In the mouse, orthologs of genes associated with the syndrome were expressed in podocytes (77). Among the fly orthologs, *sns*, *kin of irre* (*kirre*), and *cubn* have been shown to have key functions in fly nephrocytes (5). The fly orthologs of human *Nphs1* and *Kirrel1*, *sns*, and *kirre*, direct adhesion, fusion, and formation of a slit diaphragm-like structure in insect nephrocytes (25). Knockdown of *sns* or *kirre* leads to a dramatic decrease in uptake of large proteins, consistent with the role of the slit diaphragm in mammalian podocytes (21). Finally, the fly orthologs of two genes associated with nephrolithiasis, *ATPase H+ Transporting V1 Subunit B1* (*ATP6V1B1*) and *ATP6V0A4* (*Vacuolar H+-ATPase 55kD subunit* [*Vha55*] and *Vha100-2* in flies), are highly expressed in Malpighian tubule principal cells. Mutations in *ATP6V1B1* and *ATP6V0A4* have been identified in calcium oxalate kidney stone patients, suggesting that they are essential for calcium oxalate kidney stone formation (78). In the mouse, the orthologs of these two genes are hallmarks of intercalated cells, and one type of intercalated cell (intercalated type non-A non-B cell of nephron connecting tubule) matched with fly initial and transitional principal cells (Fig. 5*A*). Interestingly, flies with knockdown of *Vha55* or *Vha100-2* in the Malpighian tubule also develop calcium oxalate kidney stones (79). Altogether, the analysis of the expression of fly orthologs of human kidney disease-associated genes at the single-cell level will help develop more accurate fly models of human kidney diseases.

## Discussion

In this study, we surveyed the cell types of the adult fly kidney using snRNA-seq and identified all known cell types and their subtypes. Our data provide insights on the role in kidney differentiation and function of three previously uncharacterized TFs (*fru*, *Sba*, and *Hnf4*). In addition, we identified four cell shape regulators: *RhoGEF64c*, *Prip*, *Frq2*, and *CG10939*. We also performed a developmental trajectory analysis of principal cells, analyzed the similarity between renal stem cells and intestinal stem cells, characterized cell-to-cell communication between clusters and the metabolic differences between clusters (detailed information in *SI Appendix*, *Supplementary Text* and Figs. S2 and S11–S13). Interestingly, six clusters of principal cells mapped to different regions of the tubule and we could associate them with different physiological functions (detailed information in *SI Appendix*, *Supplementary Text* and Fig. S2). Of particular interest, we find that renal stem cells contain two clusters distinguishable by expression of *Dl^+^ klu^−^* and *Dl^−^ klu^+^* (detailed information in *SI Appendix*, *Supplementary Text* and Fig. S11), reminiscent of intestinal stem cells/enteroblasts in the midgut (29). In addition, we used FlyPhoneDB (80) to predict ligand–receptor interactions between different cell clusters, a resource that will help analyze communication among kidney cells (detailed information in *SI Appendix*, *Supplementary Text* and Fig. S12).

Additionally, our analyses of the trajectories, TFs, cell–cell communication, and metabolic pathway enrichment, provide a number of functional insights. For example, cell–cell communication analysis showed that the Notch (N) ligand only has interaction within renal stem cells and does not pair with other cell clusters (*SI Appendix*, Fig. S12*A*). Consistently, the regulons identified by SCENIC also showed that *N* is the target gene of *esg* (*SI Appendix*, Fig. S3*B*), which is consistent with previous studies showing that differential Notch activity is required for renal stem cells homeostasis, and that damage activates Notch signaling, which in turn regulates differentiation of renal stem cells to principal cells (16, 81). Renal stem cells, located in lower ureter principal cells, upper ureter principal cells, lower tubule principal cells, and lower segment principal cells can replace cells in these locations following damage (16). Furthermore, trajectory analyses demonstrated the gradual transition from cells in lower ureter principal cells, upper ureter principal cells, lower tubule principal cells, and lower segment principal cells to main segment principal cells, and initial and transitional principal cells (*SI Appendix*, Fig. S2*D*), while the metabolic enrichment analysis showed that *ry/uro/CG30016* were up-regulated in main segment principal cells, which is consistent with uric acid metabolism being the main function of renal cells. Thus, our trajectory analyses, regulons (TFs), cell–cell communication, and metabolic pathway enrichments provide insights on the molecular mechanisms of renal function.

Several studies have compared human and mouse scRNA-seq datasets in a systematic way (82, 83), which is relatively easy to do because of their close evolutionary distance. However, such analysis is more challenging with distant species due to ortholog mapping. Our cross-species analysis not only provided information about the potential functions of unknown cell types, but also gave us a better comparative understanding of kidney cells from lower species (planaria) to higher species (mouse). For example, fly lower segment principal cells map to mouse principal cells of the inner medullary collecting duct type 1/2 (Fig. 5*A*), but there is no corresponding cell type in planaria. Results of the cross-species analysis will facilitate study of the functions of specific cell types found in higher animals using lower species as models.

*Drosophila* Malpighian tubules and nephrocytes have been used successfully to model human kidney diseases. For example, mutations in the vacuolar-type H^+^-ATPase (V-ATPase) subunit genes ATP6V1B1 and ATP6V0A4 in humans have been identified in recurrent calcium oxalate kidney stones (78) and knockdown of the fly orthologs, *Vha55* and *Vha100-2*, using Uro-Gal4 led to increased formation of calcium oxalate stones in Malpighian tubules (79). Our fly kidney cell atlas will facilitate disease modeling and analysis. First, it will help narrow down the number of genes to be tested in specific cell types, as our snRNA-seq has identified cell-type–specific transcriptomes. Second, as we were able to match cell types between the fly and mouse, we are now able to associate human kidney disease-associated genes with specific fly kidney cell types. This critical information should facilitate the development of more accurate fly models of human kidney diseases.

## Materials and Methods

Details on the single-nucleus isolation and sequencing, dataset processing and bioinformatics analysis, fly genetics, immunostaining, and confocal microscopy can be found in *SI Appendix*, *SI Materials and Methods*.

## Supplementary Material

Supplementary File

Supplementary File

Supplementary File

Supplementary File

Supplementary File

Supplementary File

Supplementary File

Supplementary File

## Data Availability

All data supporting the findings of this study are available within the main text and *SI Appendix* files or from the corresponding author upon reasonable request. Raw snRNA-seq reads have been deposited in the Gene Expression Omnibus (GEO) database, https://www.ncbi.nlm.nih.gov/geo (accession no. GSE202575). Processed datasets can be mined through a web tool (https://www.flyrnai.org/scRNA/kidney/) that allows users to explore genes and cell types of interest.
