# Supplementary material for: Transcriptional and functional motifs defining renal function revealed by single-nucleus RNA sequencing
Source: Proc Natl Acad Sci U S A. 2022 Jun 13;119(25):e2203179119. doi: 10.1073/pnas.2203179119 (PMC9231607; doi:10.1073/pnas.2203179119)
Supplement: Supplementary File [file pnas.2203179119.sapp.pdf]

Supplementary Information for

Transcriptional and functional motifs defining renal function revealed by single nuclei RNA sequencing

Jun Xu[a, 1], Yifang Liu[a, 1], Hongjie Li[b,i], Alexander J. Tarashansky[c,d], Colin H. Kalicki[c], Ruei-Jiun Hung[a], Yanhui Hu[a], Aram Comjean[a], Sai Saroja Kolluru[c,d], Bo Wang[c,e], Stephen R Quake[c,d], Liqun Luo[b], Andrew P. McMahon[f], Julian A.T. Dow[g], Norbert Perrimon[a,h, 2]

[a]Department of Genetics, Blavatnik Institute, Harvard Medical School, Harvard University, Boston, MA 02115, USA

[b]Department of Biology, Howard Hughes Medical Institute, Stanford University, Stanford, CA, USA

[c]Department of Bioengineering, Stanford University, Stanford, CA, USA

[d]Chan Zuckerberg Biohub, San Francisco, CA, USA

[e]Department of Developmental Biology, Stanford University School of Medicine, Stanford, CA, USA

[f]Department of Stem Cell Biology and Regenerative Medicine, Eli and Edythe Broad Center for Regenerative Medicine and Stem Cell Research, Keck School of Medicine of the University of Southern California, Los Angeles, CA 90089, USA

[g]Institute of Molecular, Cell & Systems Biology, College of Medical, Veterinary and Life Sciences, University of Glasgow, UK

[h]Howard Hughes Medical Institute, Boston, MA, USA

[i]Present Address: Huffington Center on Aging, Department of Molecular and Human Genetics, Baylor College of Medicine, Houston, TX 77030, USA

[1]co-first authors

[2]Correspondence: perrimon@genetics.med.harvard.edu

This PDF file includes:

SI Materials and Methods
Supplementary text
Figures S1 to S13
SI References
Legends for Datasets S1 to S10

SI Materials and Methods

Single nucleus isolation and sequencing. *Drosophila* Malpighian tubules and nephrocytes were dissociated to single nuclei as previously described (1) with a few modifications. Malpighian tubules were dissected under a microscope from 5-7-day old *Drip-Gal4>GFP.nls* male and female adult flies. Nephrocytes were dissected under a fluorescence microscope from 5-7-day old *Dot-Gal4>GFP.nls* male and female adult flies. Ten flies at a time were dissected and samples immediately transferred into 1.5 ml EP tube with Schneider's medium on ice to avoid exposing the tissues to room temperature for a long period of time. Once 50 flies were dissected, EP tubes were sealed with parafilm and put on dry ice. In total, 150 male and 150 female tissues were dissected. Next steps involved spraying 100% ethanol to the dry ice near the tube to quickly freeze the sample and storing samples at -80°C for long-term. After dissection, samples were spined down (thaw samples from - 80°C) in 100 ul Schneider's medium using a bench top spinner, medium was discarded, and 100 ul homogenization butter was added (1). Subsequently, 900 ul homogenization buffer was added, and 1000ul homogenized sample transferred into the 1 ml dounce. Nuclei were released by 15-20 strokes with loose pestle and 15-20 tight pestle on ice. 1000 ul sample was filtered through 5ml cell strainer (35 um), and then filter sample using 40 um Flowmi into 1.5ml EP tube. Centrifuge for 10 min at 1000 g at 4°C. Resuspend in 1000 ul PBS/0.5% BSA with RNase inhibitor. And filter sample using 40 um Flowmi into a new EP tube. Hoechst 33342 was used to stain nuclei for more than 5 min. Then FACS and collect single nuclei into a tube for 10x Genomics.

Ten thousand nuclei were targeted for each sample when loaded into the Chromium Controller (10X Genomics, PN-120223) on a Chromium Single Cell B Chip (10X Genomics, PN-120262), and processed to generate single cell gel beads in the emulsion (GEM) according to the manufacturer's protocol (10X Genomics, CG000183). The library was generated using the Chromium Single Cell 3′ Reagent Kits v3.1 (10X Genomics, PN-1000121) and Chromium i7 Multiplex Kit (10X Genomics, PN-120262) according to the manufacturer's manual. Quality control for constructed library was performed by Agilent Bioanalyzer High Sensitivity DNA kit (Agilent Technologies, 5067-4626) for qualitative analysis. Quantification analysis was performed by Illumina Library Quantification Kit (KAPA Biosystems, KK4824). The library was sequenced on an Illumina NovaSeq system or Nextseq 500 instrument.

Dataset processing. The quality of the raw sequencing data was checked by FastQC software. The raw sequencing data were processed by cellranger count pipeline to generate the single cell matrix for each sample. The single cell matrix was analyzed by the Seurat package and Harmony was used for batch correction. The Malpighian tubules and nephrocytes samples were processed and clustered separately, and then were merged by filtering out unrelated cell clusters. The processed result of the gene expression matrix was used for the downstream data analysis.

To facilitate mining of the datasets, we developed a visualization web portal (https://www.flyrnai.org/scRNA/kidney/) that allows users to query the expression of any genes of interest in different cell types and to compare the expression of any 2 genes in individual cells. This Malpighian tubule dataset can also be mined at Fly Cell Atlas (https://flycellatlas.org/) along with other datasets generated by the FCA consortium (1).

Gene ontology (GO) analysis. Gene ontology (GO) analysis was performed by clusterProfiler. The marker genes identified using Seurat were used for GO analysis. The strength of enrichment was calculated as negative of log10(p-value), which was used to plot the barplot.

Cross-tissue analysis. Cross-tissue analysis data are from the Malpighian tubules processed dataset and midgut dataset (accession code: GSE120537). Before merging the two datasets, the number of Malpighian tubules cells was downsampled to the same size as the midgut dataset. The top markers of renal stem cells were calculated by comparing the renal stem cells with the rest of the merged dataset without intestinal stem cells. The top markers of intestinal stem cells were calculated by comparing the intestinal stem cells with the rest of the merged dataset without renal stem cells. Results of the comparison are visualized on a Venn diagram.

Pseudotemporal ordering of cells using Monocle3. Processed Malpighian tubules dataset was analyzed using Monocle3 for pseudotemporal ordering. The state representing lower ureter principal cells was chosen as the starting time point. The Ridge plot was generated by extracting the cell clustering and pseudotime information and then visualized by Seurat RidgePlot function. The gene expression heatmap was generated by merging the cells into bins by the order of pseudotime and visualized by the pheatmap R package. This analysis was done for both fly data and mouse data (GSE129798).

Transcription factors enrichment and SCENIC analysis. The top markers for each cluster were used as the candidates for TFs enrichment analysis. The markers were filtered by fold change > 3 and adjusted p-value < 0.05. TFs from the filtered markers were visualized by the Seurat DoHeatmap. The analysis of regulon activity was conducted using the SCENIC pipeline (2). Cells from the previously processed dataset were selected as the input cells. The TF and co-expressed genes were constructed by

GRNBoost2. The TF co-expression gene sets were filtered by the RcisTarget fly database. The regulon activity score AUC (Area Under the Curve) was calculated by AUCell, and the active regulons were determined by the AUCell default parameters. The regulon activity was visualized by the average AUC score for each cluster. These analyses were done for both fly data and mouse data (GSE129798).

Cell-cell communication analysis. The cell-cell communication analysis was performed using FlyPhoneDB (3) for fly data. The previously processed gene expression matrix and cell clustering information were used as the input for the analysis. Ligand-receptor interaction scores and specificity were then calculated. Cell communication at the signaling pathway level was visualized by a circle plot. The interaction of ligand-receptor pairs between two cell types was visualized by dot plot. The network was generated based on the MIST database and TF2TG literature using Cytoscape (4, 5). CellChat was used to analyze cell-cell communication of the mouse kidney data (6). The gene expression matrix and cell clustering information were obtained from GEO (GSE129798). The probability scores and p-values for ligand-receptor interactions were then calculated. Cell communication at the signaling pathway level was visualized by a circle plot and were compared to the fly results.

Cross-species analysis. Datasets included in the cross-species analysis were the processed dataset from this study for the fly, the mouse kidney dataset GSE129798 and the planarian dataset GSE111764. The analysis was conducted using the SAMap software (7). The input file for SAMap was processed using the Self-Assembling-Manifold (SAM) algorithm. Alignments for each cell type in fly and mouse were calculated by the get_mapping_scores function. Enriched gene pairs from the aligned cell types were retrieved by find_all function with a default alignment score threshold of 0.1. The SAMap results were visualized by the sankey_plot function. To assess the specificity of cluster mapping between fly Malpighian tubules and mouse kidney, the top markers of each cell type from mouse kidney data were also compared to the top markers of the FCA 252 cell clusters representing all fly tissues (1) using DRscDB (8).

Fly genetics. Fly husbandry and crosses were performed under a 12:12 hour light:dark photoperiod at 25°C. *Hand-GFP; 4xHand-Gal4/CyO* and *Dot-Gal4* stocks are gifts from Dr. Han Zhe. *esg-Gal4* and *Pros-Gal4* are from the Perrimon lab stock collection.

The following strains were obtained from the Bloomington *Drosophila* Stock Center: *Drip-Gal4* (BL66782), *UAS-GFP.nls* (BL4776), *UAS-mCD8::RFP* (BL32219), *UAS-mCD8::GFP* (BL32185), *c42-Gal4* (BL30835), *Uro-Gal4; tubGal80ts* (BL91415), *Alp4-Gal4* (BL30840), *SPR-Gal4* (BL84692), *wnt4-Gal4* (BL67449), *Esyt2-Gal4* (BL77712), *Debcl-Gal4* (BL81163), *ih-Gal4* (BL76162), *CG30377-Gal4* (BL67426), *Octα2R-Gal4* (BL67637), *tsh-Gal4* (BL3040), *fru-Gal4* (BL30027), *Sba-Gal4* (BL67640), *Doc2-Gal4* (BL26436), *Lim3-Gal4* (BL67450), *Pvf1-Gal4* (BL23032), *Hnf4-GFP-FLAG* (BL38649), *y v; UAS-Luc-RNAi, attp2* (BL31603), *y v; UAS-wat-RNAi, attp40* (BL67801), *y v; UAS-tutl-RNAi, attp40* (BL54850), *y v; UAS-axed-RNAi, attp40*

(BL62928), *y v; UAS-prip-RNAi, attp40* (BL50695), *y v; UAS-prip-RNAi, attp2* (BL44464), *y v; UAS-nep2-RNAi, attp40* (BL61902), *y v; UAS-CG13323-RNAi, attp40* (BL53969), *y v; UAS-Lgr1-RNAi, attp2* (BL51465), *y v; UAS-Octα2R-RNAi, attp2* (BL50678), *y v; UAS-CG30377-RNAi, attp40* (BL51386), *y v; UAS-notum-RNAi, attp40* (BL55379), *y v; UAS-TkR99D-RNAi, attp2* (BL27513), *y v; UAS-ih-RNAi, attp40* (BL58089), *y v; UAS-ncc69-RNAi, attp2* (BL28682), *y v; UAS-frq2-RNAi, attp2* (BL28711), *y v; UAS-qvr-RNAi, attp40* (BL58061), *y v; UAS-CG10939-RNAi, attp40* (BL65156), *y v; UAS-CG42594-RNAi, attp2* (BL35006), *y v; UAS-RhoGEF64c-RNAi, attp2* (BL31130), *y v; UAS-hnf4-RNAi, attp2* (BL29375), *y v; UAS-Lim3-RNAi, attp2* (BL26227).

The following strains were obtained from the Vienna *Drosophila* Resource Center: *y w (1118)*; *attp landing site* (v60100), *UAS-RhoGEF64c-RNAi* (v47121), *UAS-RhoGEF64c-RNAi* (v105252), *y v; UAS-fru-RNAi, attp40* (v330035).

For the screen shown in Fig. S4B, 8 *tsh-Gal4/CyO; UAS-mCD8::GFP* virgin females were crossed with 4 RNAi males. Flies were raised at 22°C and the ratio of Cy+/Cy was determined.

Immunostaining and confocal microscopy. *Drosophila* Malpighian tubules (and guts), pericardial nephrocytes (included in the whole abdomen) and garland nephrocyte cells (and foreguts and crops) from adult females were fixed in 4% paraformaldehyde in Phosphate-buffered saline (PBS) at room temperature for 1 hour, incubated for 1 hour in Blocking Buffer (5% normal donkey serum, 0.3% Triton X-100, 0.1% bovine serum albumin (BSA) in PBS), and stained with primary antibodies overnight at 4°C in PBST (0.3% Triton X-100, 0.1% BSA in PBS). Primary antibodies and their dilutions used were: mouse anti-GFP (Invitrogen, A11120; 1:300) and mouse anti-discs-large (DSHB, 4F3,1:50). After primary antibody incubation, the tissues were washed 3 times with PBST, stained with 4′,6-diamidino-2-phenylindole (DAPI) (1:2000 dilution), Phalloidin TRITC (Sigma-Aldrich, 1:2000) and Alexa Fluor-conjugated donkey-anti-mouse (Molecular Probes, 1:1000), in PBST at 22°C for 2 hours, washed 3 times with PBST, and mounted in Vectashield medium.

All images presented in this study are confocal images captured with a Nikon Ti2 Spinning Disk confocal microscope. Z-stacks of 15-20 images covering one layer of the epithelium from the apical to the basal side were obtained, adjusted, and assembled using NIH Fiji (ImageJ), and shown as a maximum projection. Details of the imaging method are as follows: Samples were imaged with a Yokogawa CSU-W1 single disk (50 μm pinhole size) spinning disk confocal unit attached to a fully motorized Nikon Ti2 inverted microscope equipped with a Nikon linear-encoded motorized stage with a Mad City Labs 500 μm range Nano-Drive Z piezo insert, an Andor Zyla 4.2 plus (6.5 μm photodiode size) sCMOS camera using a Nikon Plan Apo 60x/1.4 NA DIC oil immersion objective lens with Cargille Type 37 immersion oil (cultured cells) or a Nikon Plan Apo 20x/0.75 DIC air objective lens (tissue samples). The final digital resolution of the image was 0.109 and 0.325 μm/pixel, respectively. Fluorescence from DAPI, Alexa Fluor (AF)-488, and AF555 was collected by illuminating the sample with directly modulated solid-state lasers 405 nm diode 100 mW (at the fiber tip) laser line, 488 nm

diode 100 mW laser line, and 561 nm DPSS 100 mW laser line in a Toptica iChrome MLE laser combiner, respectively. A hard-coated Semrock Di01-T405/488/568/647 multi-bandpass dichroic mirror was used for all channels. Signal from each channel was acquired sequentially with hard-coated Chroma ET455/50, Chroma ET525/36 nm, and Chroma ET605/52 nm emission filters in a filter wheel placed within the scan unit, for blue, green, and red channels, respectively. Nikon Elements AR 5.02 acquisition software was used to acquire the data. 2 µm range Z-stacks, set by indicating the middle focal plane and a z-step interval of 50 µm, were acquired using piezo Z-device, with the shutter closed during axial movement. Images were acquired by collecting the entire Z-stack in each color or by acquiring each channel in each focal plane within the Z stack. Data were saved as ND2 files.

Lipid and Carbohydrate Measurements. We measured fly carbohydrates and triglycerides as described previously (9, 10). To prepare fly lysates for metabolic assays, we homogenized 4 flies from each group with 300 µl PBS supplemented with 0.2% Triton X-100 and heated at 70°C for 10 min. The supernatant was collected after centrifugation at 3000g for 1 min at 4°C. 10 µl of supernatant was used for protein quantification using Bradford Reagent (Sigma, B6916-500ML). Whole-body trehalose levels were measured from 10 µl of supernatant treated with 0.2 µl trehalase (Megazyme; E-TREH) at 37°C for 30 min using glucose assay reagent (Megazyme; K-GLUC) following the manufacturer’s protocol. Whole-body glycogen levels were determined from 10 µl of supernatant preincubated with 1 µl amyloglucosidase (Sigma-Aldrich; A7420) at 37°C for 30 min using glucose assay reagent (Megazyme; K-GLUC). We subtracted the amount of free glucose from the measurement and then normalized the subtracted values to protein levels in the supernatant. To measure whole body triglycerides, we processed 10 µl of supernatant using a Serum Triglyceride Determination kit (Sigma, TR0100). We subtracted the amount of free glycerol from the measurement and then normalized the subtracted values to protein levels.

mRNA quantification. Total RNA was extracted from 25 Malpighian tubules using NucleoSpin RNA kit (Macherey-Nagel) and converted to cDNA using the iScript cDNA Synthesis kit (Bio-Rad). cDNAs were analyzed by quantitative PCR (qPCR) using the SYBR Green kit (Bio-Rad) and Bio-Rad CFX Manager software. *rp49* were used as internal control. Each RT-qPCR was performed with three technical replicates and three biological replicates. qPCR primer pairs (forward & reverse) are shown below:
RhoGEF64C: GAAGCGGATTGGGATGATTA & CGTGGTACTGCAGCTGTGTG
rp49: ATCGGTTACGGATCGAACAA & GACAATCTCCTTGCGCTTCT

Supplementary text

Developmental trajectory analysis of principal cells. Principal cells (PCs) are mitochondria-rich and transport protons through an apical, plasma membrane vacuolar H^+-ATPase (V-ATPase) (11). The main functions of principal cells are to set up a potassium gradient (12, 13), which enters the cell basolaterally through the combined activity of Na^+, K^+-ATPase (14), inward rectifier potassium channels (15-17), and

potassium cotransports (18-20). We identified six PC clusters from the scRNA-seq dataset and identified Gal4 lines that allowed us to precisely map their anatomical locations (Fig. 1A). To understand the functional differences of each PC cell cluster, we performed a GO analysis based on marker genes (Fig. S2A). Most of the GO terms refer to transport and responses to toxic substances, reflecting the main functions of the tubule. Interestingly, the top 10 terms in lower tubule PCs refer to transport, suggesting that the function of lower tubule principal cells is to transport substances between Malpighian tubules and the hemolymph (GO information is in Dataset S8).

scRNA-seq enables the exploration of the continuous differentiation trajectory of a developmental process. Thus, to analyze the developmental trajectory of principal cells, we conducted a pseudotime analysis by ordering cells along a reconstructed trajectory using Monocle3 (Fig. S2B and S2C). Consistent with the distribution distance on the UMAP, inferred trajectories demonstrated gradual transitions from cells in lower ureter principal cells, upper ureter principal cells, lower tubule principal cells, and lower segment principal cells to main segment principal cells, initial and transitional principal cellss (Fig. S2D). On the UMAP, lower segment principal cells are close to lower ureter principal cells, upper ureter principal cells, and lower tubule principal cells. The pseudotime analysis also showed that the state of lower segment principal cells is a co-mixture of lower ureter principal cells, upper ureter principal cells, and lower tubule principal cells (Fig. S2D). These results are consistent with our observation *in vivo* using *Esyt2-Gal4* (Fig. 1C), which suggested that lower segment PCs represent a new cell cluster that is different from previously reported *Alp4* expressed cells.

We chose *Best2*, *bifid (bi), Sarcoplasmic calcium-binding protein 2* (*Scp2*), *PDGF- and VEGF-receptor related* (*Pvr*), *Uro*, *salty dog* (*salt)*, *Alp4*, *SPR*, and *Transient receptor potential cation channel A1* (*TrpA1*) as representative genes for each cluster (Fig. S2E). A survey of our scRNA-seq dataset revealed that the expression of *TrpA1* gradually decreased along the pseudotime, followed by increased transcription of *Alp4* and *SPR*. The expression of *Pvr*, *Uro* and *salt* was elevated at the more geographical distant region of main segment principal cells, while the progressive increase of *Best2*, *bi*, and *Scp2* expression was only observed in initial and transitional principal cells (Fig. S2E). These results indicate that the patterns of expression of marker genes in each cluster are in concordance with the pseudotime analysis of the clusters.

Similarities between renal and intestinal stem cells. Compared to a previous report that performed scRNAseq of the Malpighian tubules focusing on the stem cell zone (21), our study captured more cells and more cell types. Wang and Spradling focused on the response of renal stem cells to tissue injury. Renal stem cells were previously identified as a distinct population that expresses *esg* (22). Renal stem cells are located in the lower ureter, the upper ureter and lower segment of the Malpighian tubule with small nuclei (23). They respond to tissue injury by upregulating the JNK, EGFR/MAPK, Hippo/Yki and JAK/STAT pathways that promote renal stem cells daughter differentiation (21). Renal stem cells originate from the same pool of adult midgut progenitors that generate the posterior midgut intestinal stem cells (23, 24). To examine how similar renal stem cells are to intestinal stem cells, we compared the

snRNA-seq renal stem cells data with previously reported scRNAseq intestinal stem cells data (25). Consistent with their common origin (23), the two stem cell clusters have high similarity at the gene expression level compared to other cell clusters (Fig. S11A).

The *esg* gene, a stem cell marker for both renal stem cells and intestinal stem cells (Fig. S11B and 11C), encodes a transcription factor that contributes to stem cell maintenance through modulation of Notch activity (26). In the intestine, *esg* is not only expressed in intestinal stem cells but also in AstC-EEs (enteroendocrine cells that express *Allatostatin C*, *AstC*) and NPF-EEs (EEs that express *neuropeptide F*, *NPF*) (25). In the intestine, ISCs are highly mitotic, especially during regeneration, and give rise to a transient progenitor, the enteroblast (EB) (27, 28), whereas in the Malpighian tubule, renal stem cells normally divide very slowly (19). In our previous gut scRNA-seq study, ISCs/EBs were annotated as one cluster based on the expression of *Dl* and *esg*. However, this cluster could be split into ISCs and EBs, as one subset of cells in the ISC/EB cluster is Dl^{+} klu^{-} and another subset is Dl^{-} klu^{+} (25). Interestingly, we could also identify two renal stem cells sub-clusters based on the expression of Dl^{+} klu^{-} and Dl^{-} klu^{+} (Fig. S11D and S11E). The Dl^{+} klu^{-} sub-cluster specifically expresses *Dl*, *N*, and *esg*, reminiscent to the Dl^{+} klu^{-} sub-cluster of ISC (Fig. S11F), whereas the Dl^{-} klu^{+} sub-cluster specifically expresses *E(spl)m3-HLH*, *E(spl)malpha-BFM, E(spl)mbeta-HLH*, which are transcription factors executing Notch-mediated cellular differentiation (29, 30, Fig. S11F).

Cell-cell communication networks in the fly kidney. Previous studies have indicated that the survival, renewal, and differentiation of principal cells (PCs) and stellate cells (SCs) are largely regulated through their cross-talk with renal stem cells (RSCs) (21-23). We used FlyPhoneDB (Liu et al, 2021) to explore cell–cell communication between the different fly kidney cell clusters. FlyPhoneDB was established recently and provides predictions of ligand-receptor interactions based on fly scRNA-seq data. We analyzed 13 major pathways and indicated their cell–cell interaction pairs between the different cell clusters (Fig.S12A). Strikingly, the Notch ligand only has interaction within RSCs and does not pair with other cell clusters (Fig. S12A). This is consistent with previous studies showing that differential Notch activity is required for RSC homeostasis and that damage activates Notch signaling, which in turn regulates differentiation of RSCs to PCs (21, 31). Further, we found that the EGFR signaling pathway connects RSCs and all SCs and PCs, with a preferentially strong interaction with main segment SCs and main segment PCs (Fig. S12A). These are consistent with previous studies showing that EGFR is dispensable for RSC maintenance but required for RSC proliferation (32). In addition, FlyPhoneDB predicts a strong interaction from main segment SCs to main segment PCs with the Pvf1-Pvr ligand-receptor pair (Fig. S12B). This interaction is based on the gene expression pattern in cell clusters of main segment SCs and PCs via MIST database and TF2TG literatures (Fig. S12C). Consistent with this, *Pvf1* was highly expressed in main segment SCs as detected using *Pvf1-Gal4>mCD8::GFP* (Fig. S12D). Altogether,

FlyPhoneDB predicts a number of specific signaling events between Malpighian tubule cell clusters. The full list of predicted gene pairs can be found in Dataset S9.

Metabolic pathway analysis. The basic functions of mammalian kidneys include metabolism of carbohydrates, proteins, lipids and other nutrients. As in mammalian kidneys, insect Malpighian tubules and nephrocytes play an essential role in the maintenance of ionic, acid–base and water balance, and elimination of metabolic and foreign toxins and homeostasis. To further understand metabolism in the fly kidney, we analyzed the KEGG metabolic pathways in UMAP of fly kidney snRNA-seq using AUCell software (2). Among 86 KEGG metabolic pathways, purine metabolism, glycerophospholipid metabolism, nicotinate and nicotinamide metabolism, starch and sucrose metabolism were more expressed than other pathways (Fig. S12A). A statistical significance analysis showed that 38 pathways were specifically expressed in one or two cell types (Fig. S12B). For examples, caffeine metabolism was specifically expressed in the main segment principal cells; nitrogen metabolism was specifically expressed in the main segment stellate cells; arginine and proline metabolism was specifically expressed in the lower segment principal cells and lower ureter principal cells; and folate biosynthesis was specifically expressed in the lower segment principal cells while thiamine metabolism was found in the upper ureter principal cells (Fig. S12C). Regarding purine metabolism, Xanthine oxidation is a necessary step in the catabolic pathway for purines toward urate, allantoin and urea. Dysfunction of xanthine oxidase/dehydrogenase (XO/XDH) causes a build-up of high levels of xanthine and hypoxanthine forming stones in humans and flies (33-35). Human ancestors lost the ability to synthesize a functional urate oxidase due to multiple point mutations in the *Uro* gene, resulting in increased serum and urinary uric acid (UA) levels (36). In the UA pathway, humans and flies share some of the same steps. The product of the fly *Uro* gene catalyzes formation of allantoin from UA (Fig. S13A). Most UA pathway genes are enriched in fly kidney cells, specifically in principal cells (Fig. S13B and C). The enzymes that control the last three steps, which are encoded by *rosy* (*ry*), *Uro* and *CG30016,* are highly enriched in main segment principal cells, suggesting that the last step occurs in this region (Fig. S13D). *ry* is the homolog of human XDH and loss of function of *ry* is associated with bloating in the lower tubules and formation of stones (37). Metabolomic analysis of *ry* mutants showed significant changes up to five metabolites away from the metabolic lesion, with large increases in levels of hypoxanthine and xanthine, and undetectable levels of the downstream metabolite UA (38). The product of *CG30016* is predicted to have hydroxyisourate hydrolase activity and to be involved in purine nucleobase metabolism. It will be interesting to determine whether this gene also plays a role in maintaining fly urate levels.

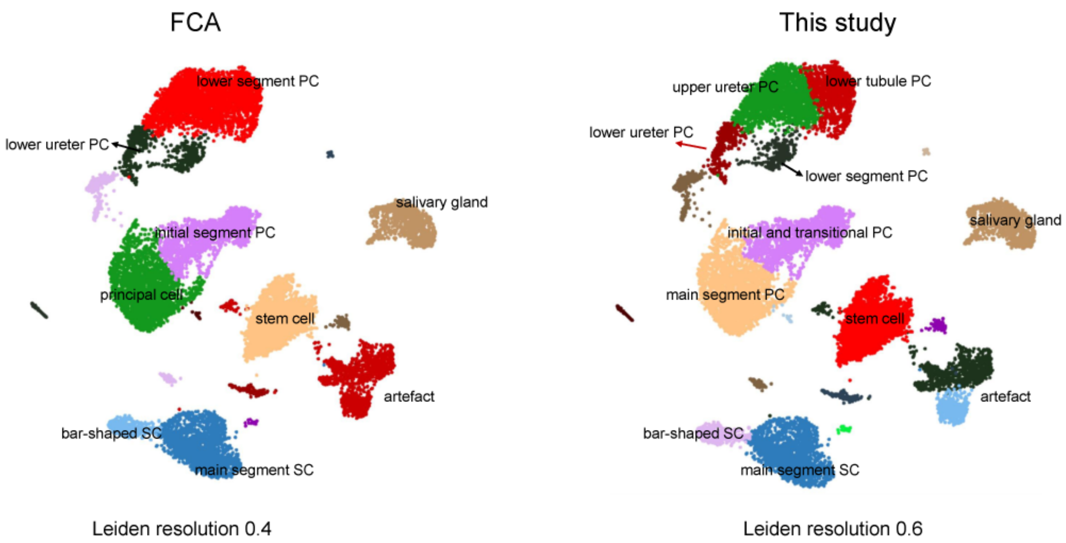

Figure S1. High resolution snRNA-seq analysis of the adult Malpighian tubule. Left, UMAP of the Malpighian tubules Fly Cell Atlas (FCA) data set at Leiden resolution 0.4 (1). Right, annotation of the same data set at Leiden resolution 0.6 (this study). The FCA analysis reports four clusters for principal cells: lower ureter PC, lower segment PC, principal cell, and initial segment PC. In this study, we defined six clusters for principal cells based on Gal4 reporter lines: lower ureter PC, lower ureter PC, lower tubule PC, lower segment PC, main segment PC, and initial segment PC. Note that this figure contains all the original clusters, including non-Malpighian tubule cell clusters (salivary glands, artefacts), which we did not include in Fig. 1A.

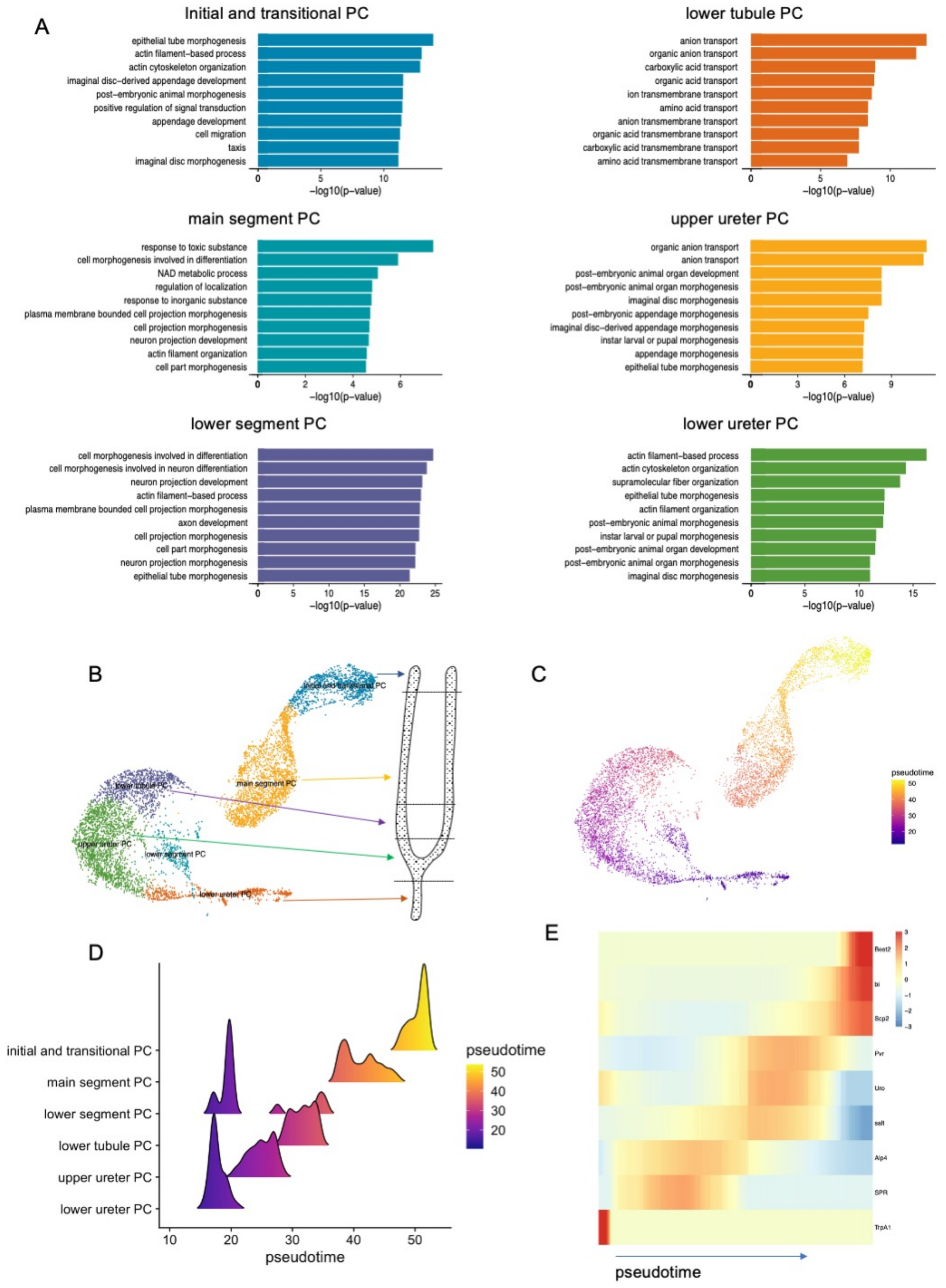

Figure S2. Pseudotime and GO analyses of the six PC sub-clusters. (A) GO analysis of each principal cell (PC) cluster. Initial and transitional PCs include epithelial tube morphogenesis, actin-filament based process, and actin cytoskeleton organization. Main segment PCs include response to toxic substance, cell morphogenesis involved in differentiation, and NAD metabolic process. Lower segment PCs include cell morphogenesis involved in differentiation, cell

morphogenesis involved in neuronal differentiation, and actin-filament based process GO terms. Lower tubule PCs include terms such as anion transport, organic anion transport, and carboxylic acid transport. Upper ureter PC terms include organic anion transport, and anion transport. Lower ureter PC GO terms include actin filament-based process, actin cytoskeleton organization, and supramolecular fiber organization. All the top 10 terms in lower tubule PCs are related to transport. The top 10 terms are displayed. (B) UMAP of PCs showing a geographical map of the tubule. (C) Cell pseudotime was inferred using Monocle3. Purple at the beginning becomes yellow over pseudotime. (D) Sub-cell type populations for each inferred cellular trajectory. The x-axis indicates the inferred pseudotime and the y-axis indicates the height of density estimated and visualized by the RidgePlot function of Seurat R package. (E) Heatmap showing gene expression patterns during differentiation along pseudotime.

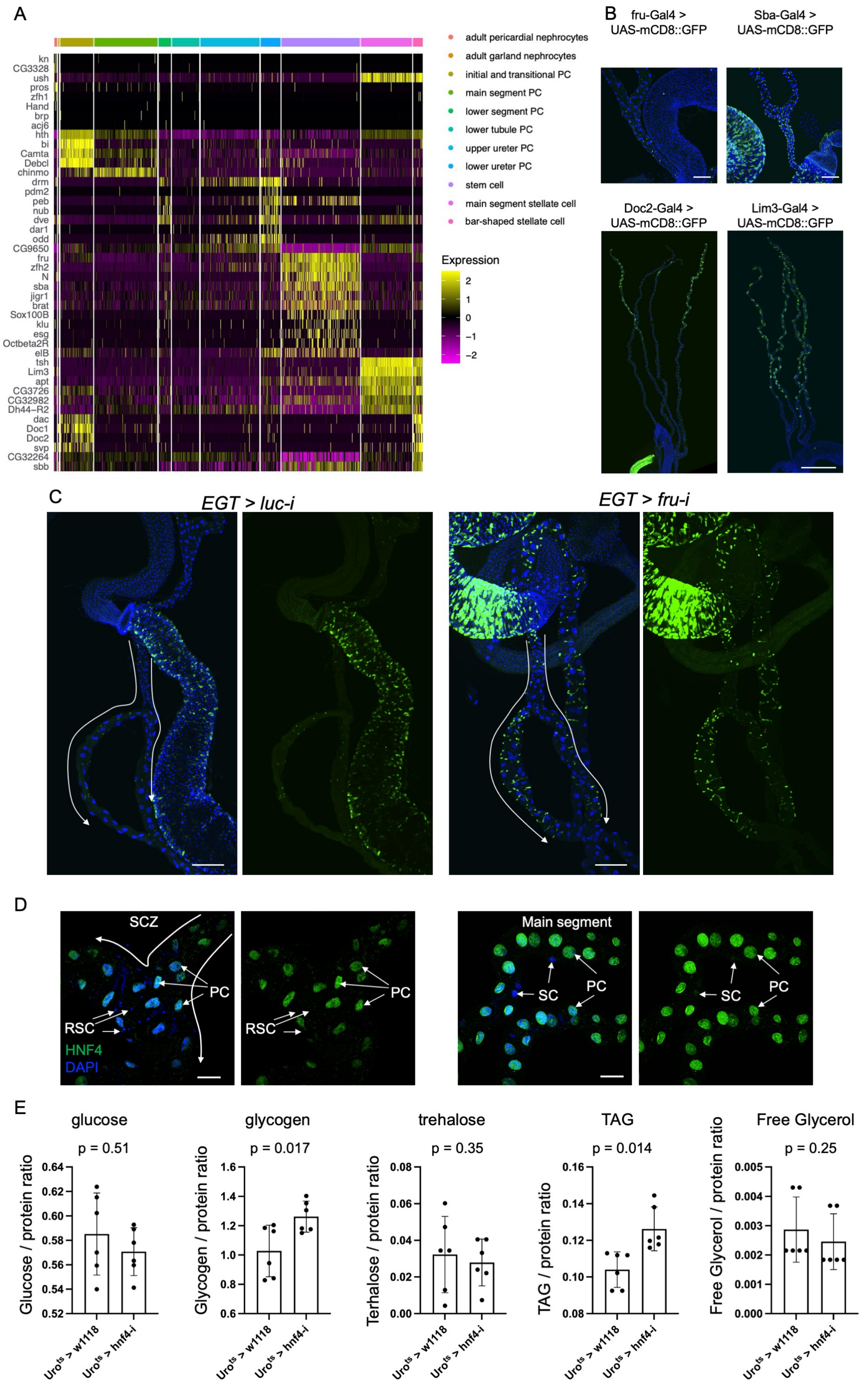
A
adult pericardial nephrocytes
adult garland nephrocytes
initial and transitional PC
main segment PC
lower segment PC
lower tubule PC
upper ureter PC
lower ureter PC
stem cell
main segment stellate cell
bar-shaped stellate cell
Expression
B
fru-Gal4 > UAS-mCD8::GFP
Sba-Gal4 > UAS-mCD8::GFP
Doc2-Gal4 > UAS-mCD8::GFP
Lim3-Gal4 > UAS-mCD8::GFP
C
EGT > luc-i
EGT > fru-i
D
SCZ
PC
RSC
HNF4
DAPI
Main segment
SC
E
glucose
p = 0.51
Glucose / protein ratio
glycogen
p = 0.017
Glycogen / protein ratio
trehalose
p = 0.35
Terhalose / protein ratio
TAG
p = 0.014
TAG / protein ratio
Free Glycerol
p = 0.25
Free Glycerol / protein ratio
Uro^ts > w1118
Uro^ts > hnf4-i

Figure S3. **Cell-type-specific gene regulatory landscape of the fly kidney.** (*A*) Heat map profile of transcription factors (TFs) in all clusters. Genes were ranked based on expression levels (fold change > 3) and adjusted p-value (<0.05) in each condition in the heat map. (*B*) Expression of *fru*, *Sba*, *Doc2* and *Lim3* visualized using fru-Gal4, Sba-Gal4, Doc2-Gal4 and Lim3-Gal4 driven UAS-CD8::GFP expression, respectively. Scale bars = 100 μm in upper lane. Scale bars = 500 μm in lower pane. (*C*) Knockdown of *fru* by using EGT induced renal stem cell proliferation. Luc RNAi or *fru* RNAi in renal stem cells for 7d. White lines show the Malpighian tubule stem cell zone. (D) *Hnf4* expression pattern in Malpighian tubules. Left shows the HNF4 staining (red fluorescence) in the stem cell zone (SCZ) and right in the main segment region. HNF4 signal is only observed in PCs, not in SCs and RSCs. DAPI stains the nuclei. (E) The whole-body level of glucose, glycogen, trehalose, TAG, Free Glycerol changes in the Uro^{ts} > *w1118* and Uro^{ts} > *Hnf4-i* are expressed ubiquitously in the principal cells for 8d.

A

♀: tsh-Gal4/*Cy*; UAS-mCD8::GFP

x

♂: TRiP-RNAi / VDRC-RNAi

↓

1. Count fly number of *Cy* or *Cy+* phenotype (confirmed by GFP)
2. Shape change of stellate cell
3. Number change of stellate cell

B

	Gene name	Identifier	*Cy+*	*Cy*	*Cy+/Cy*	Lethal stage	Shape of SC	Number of SC	Function
1	luciferase	BL31603	183	192	95.31%	-	-	-	-
2	tutl	BL54850	71	331	21.45%	early adult	-	-	Cell–cell adhesion mediator
3	prip	BL50695	63	261	24.14%	lava, embryo	Changed	-	aquaporin
4	wat	BL67801	55	200	27.50%	most die at pupa	-	-	Waterproof, liquid clearance
5	CG30377	BL51386	19	48	39.58%	lava, embryo	-	-	copper ion homeostasis
6	Lgr1	BL51465	30	74	40.54%	lava, embryo	-	-	hormone receptor
7	CG10939	BL65156	60	139	43.17%	lava, embryo	Changed	-	scaffold linking the plasma membrane
8	qvr	BL58061	65	134	48.51%	lava, embryo	-	-	GPI anchor binding
9	ih	BL58089	88	155	56.77%	lava, embryo	-	-	Potassium channel
10	axed	BL62928	123	196	62.76%	most die at pupa	-	-	neurogenesis
11	Octα2R	BL50678	83	125	66.40%	lava, embryo	-	-	octopamine receptor
12	notum	BL55379	92	135	68.15%	lava, embryo, pupa	-	-	pectinacetylesterase
13	TkR99D	BL27513	80	115	69.57%	-	-	-	tachykinin receptor
14	CG13323	BL53969	162	211	76.78%	-	-	-	unknown
15	prip	BL44464	63	75	84.00%	-	-	-	aquaporin
16	CG42594	BL35006	68	77	88.31%	-	-	-	ion leak channel
17	nep2	BL61902	144	163	88.34%	-	-	-	Peptidase
18	ncc69	BL28682	132	149	88.59%	-	-	-	sodium chloride cotransporter
19	frq2	BL28711	109	103	105.83%	-	Changed	Reduced	neuromuscular junction development
20	RhoGEF64C	BL31130	36	43	83.72%	-	-	-	regulation of Rho protein signal transduction
21	VDRC-Con	V60100	43	44	97.73%	-	-	-	-
22	RhoGEF64C-V1	V47121	4	111	3.60%	pupa, lava, embryo	Changed	Reduced	-
23	RhoGEF64C-V2	V105252	19	56	33.93%	pupa, lava, embryo	Changed	Reduced	-

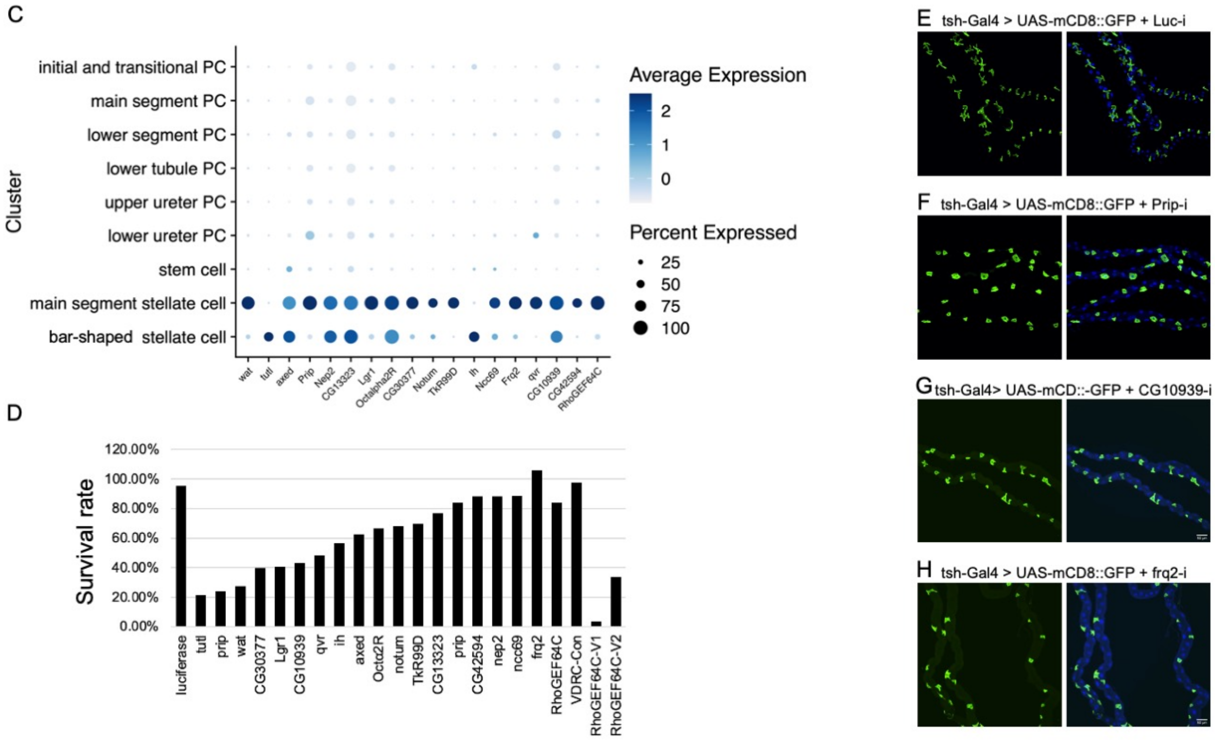

Figure S4. Phenotypes associate with the top stellate cell marker genes. (A) Screening strategy. Stellate cells (SCs) were visualized by tsh-Gal4 driving mCD8::GFP. (B) List of the genes tested in the screen, RNAi line identifiers, ratio of Cy+ versus Cy progenies, stage of lethality, effects on cell shape and number, and a short description of gene function. (C) Dot plots indicating the expression level of candidate genes in bar-shaped SCs and main segment SCs. (D) Histogram showing the survival rate. (E-H) SC cell shape phenotypes associated with RNAi knockdown of *Prip*, *CG10939* or *Frequenin 2* (*Frq2*). DAPI (blue) staining for nuclei.

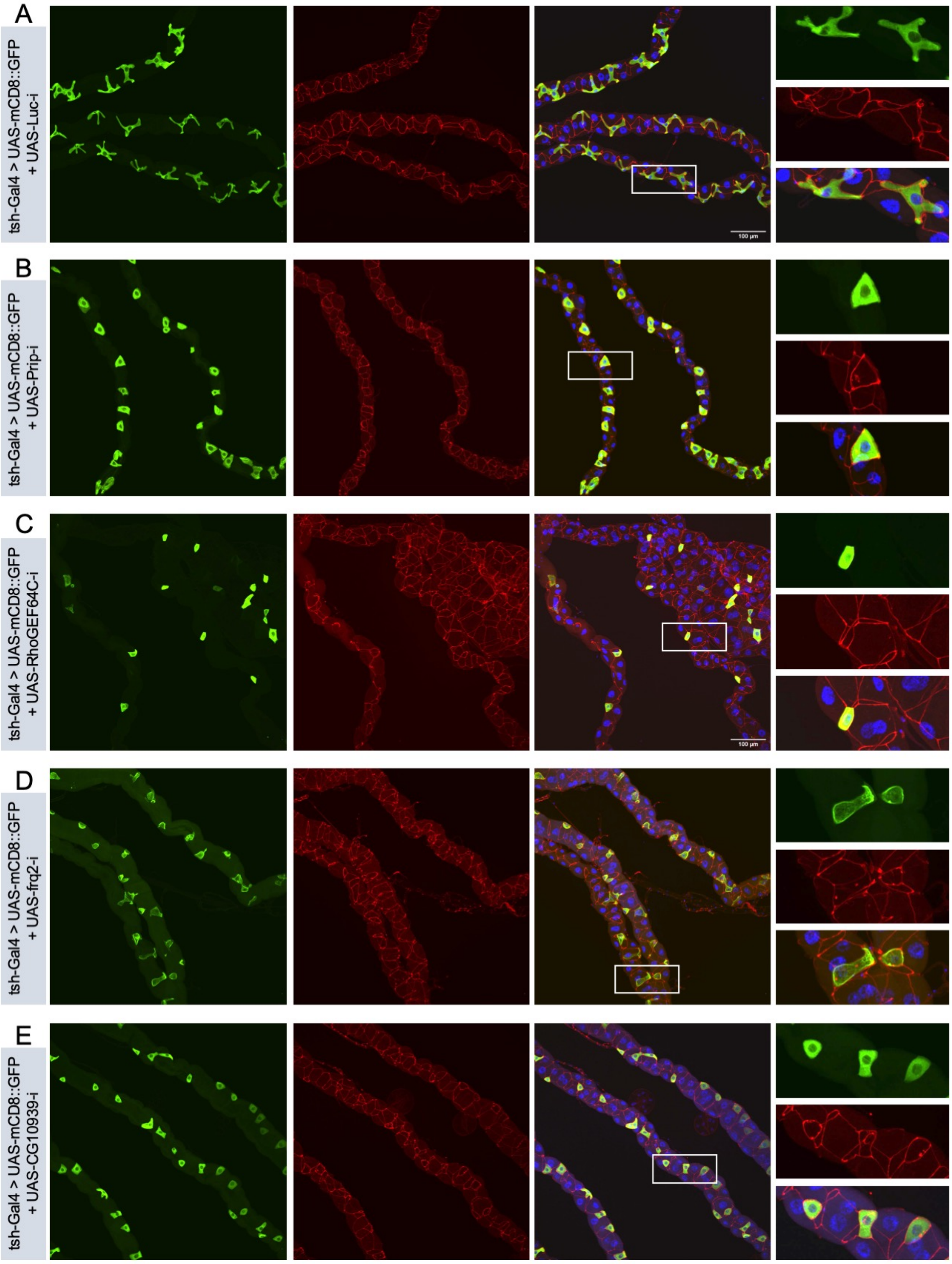

Figure S5. Morphology of septate junctions in Malpighian tubules. tsh-Gal4 drives mCD8::GFP expression in the adult Malpighian tubules in evenly spaced SCs. Septate junctions are labelled using anti-Dlg (red). White boxes indicate the zoomed-in regions. DAPI (blue) staining for nuclei. (A) *Luciferase* RNAi control. (B-E) Phenotypes associated with RNAi knockdown of *Prip*, *RhoGEF64c*, *Frq2* or *CG10939*. Scale bars = 100 µm.

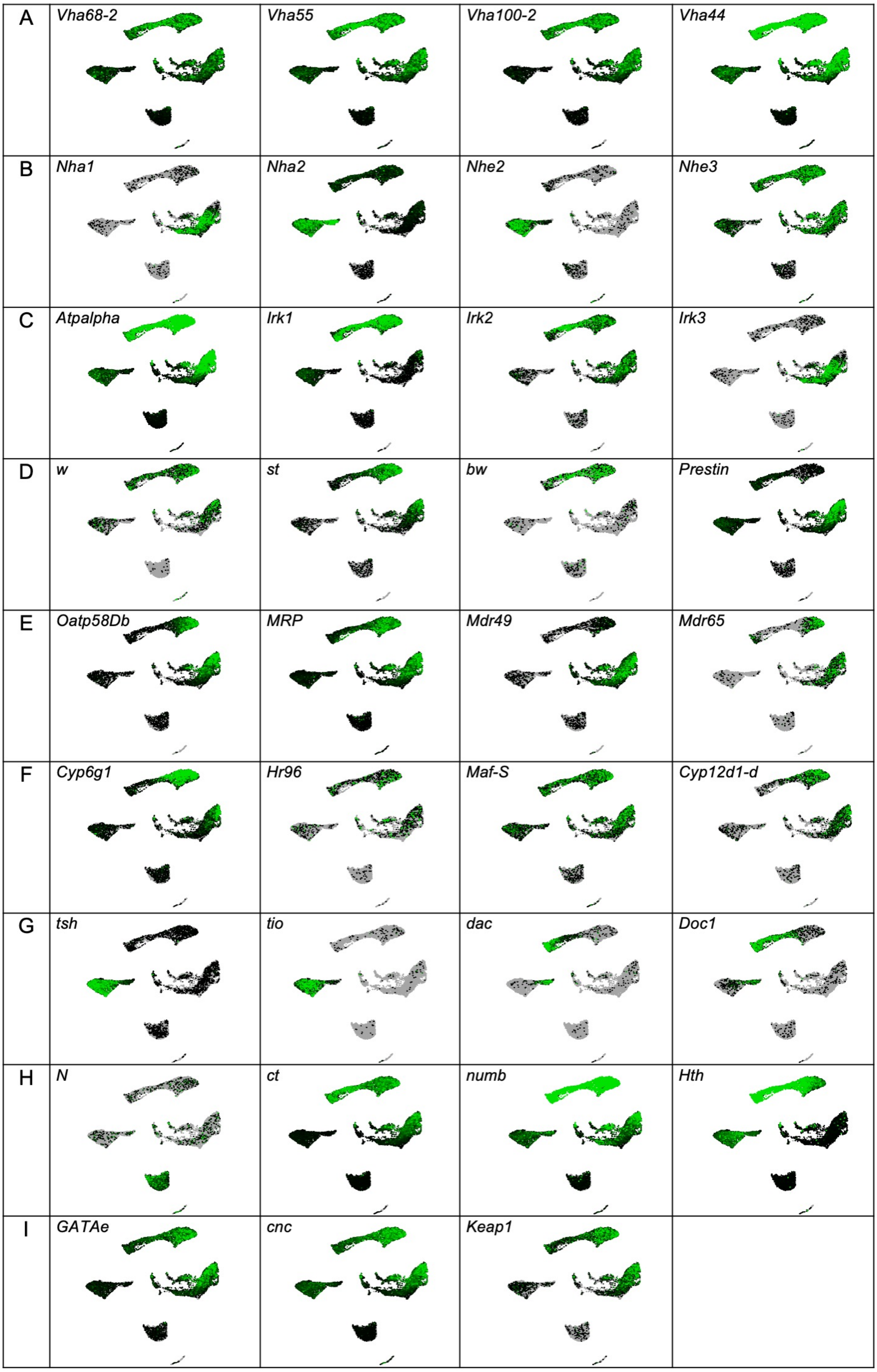

Figure S6. Gene expression in Malpighian tubules.

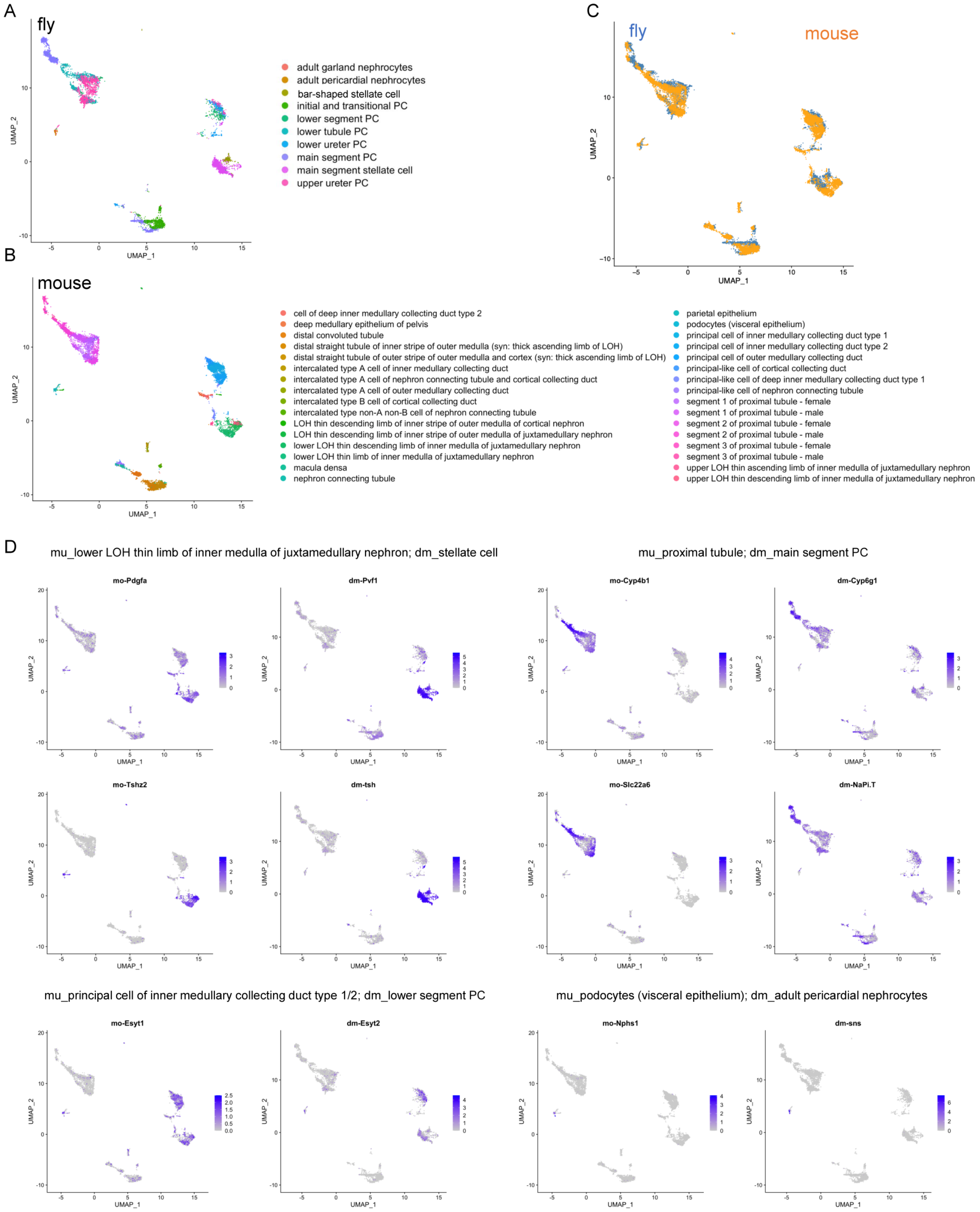

Figure S7. Cross-species analysis of fly kidney and mouse using SAMap. (A and B) Low dimensional representations of the cell atlases through homologous gene pairs in the mouse and fly using SAMap. (C) UMAP projection of the combined mouse (yellow) and fly (blue) manifolds. (D) Expression of orthologous gene pairs on the UMAP projection. Expressing cells are in blue and cells with no expression are shown in gray.

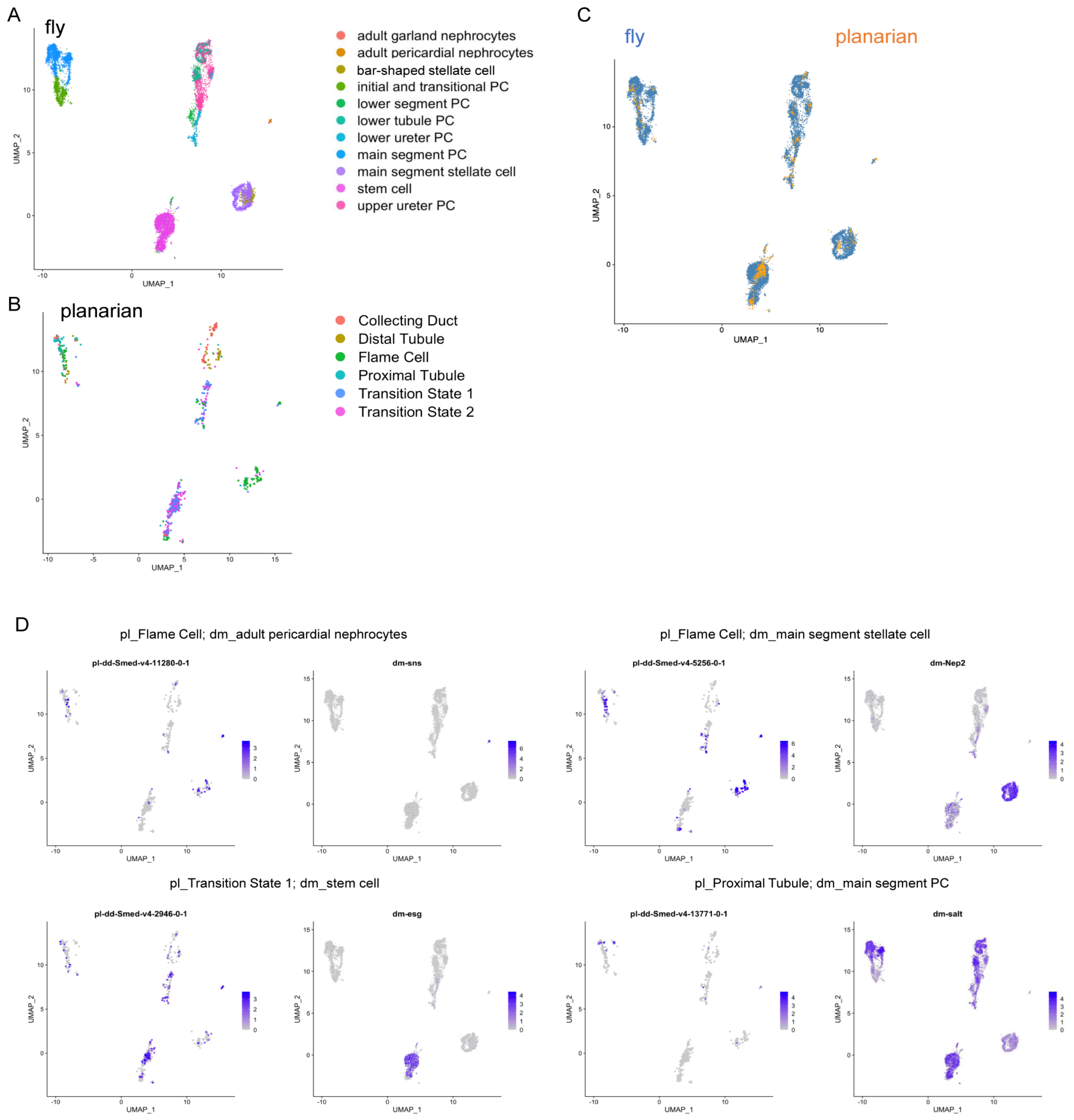

Figure S8. Cross-species analysis of fly kidney and planaria protonephridia using SAMap. (A and B) Low dimensional representations of the cell atlases through homologous gene pairs in the planaria and fly using SAMap. (C) UMAP projection of the combined planaria (yellow) and fly (blue) manifolds. (E) Expression of orthologous gene pairs on the UMAP projection. Expressing cells are in blue and cells with no expression are shown in gray.

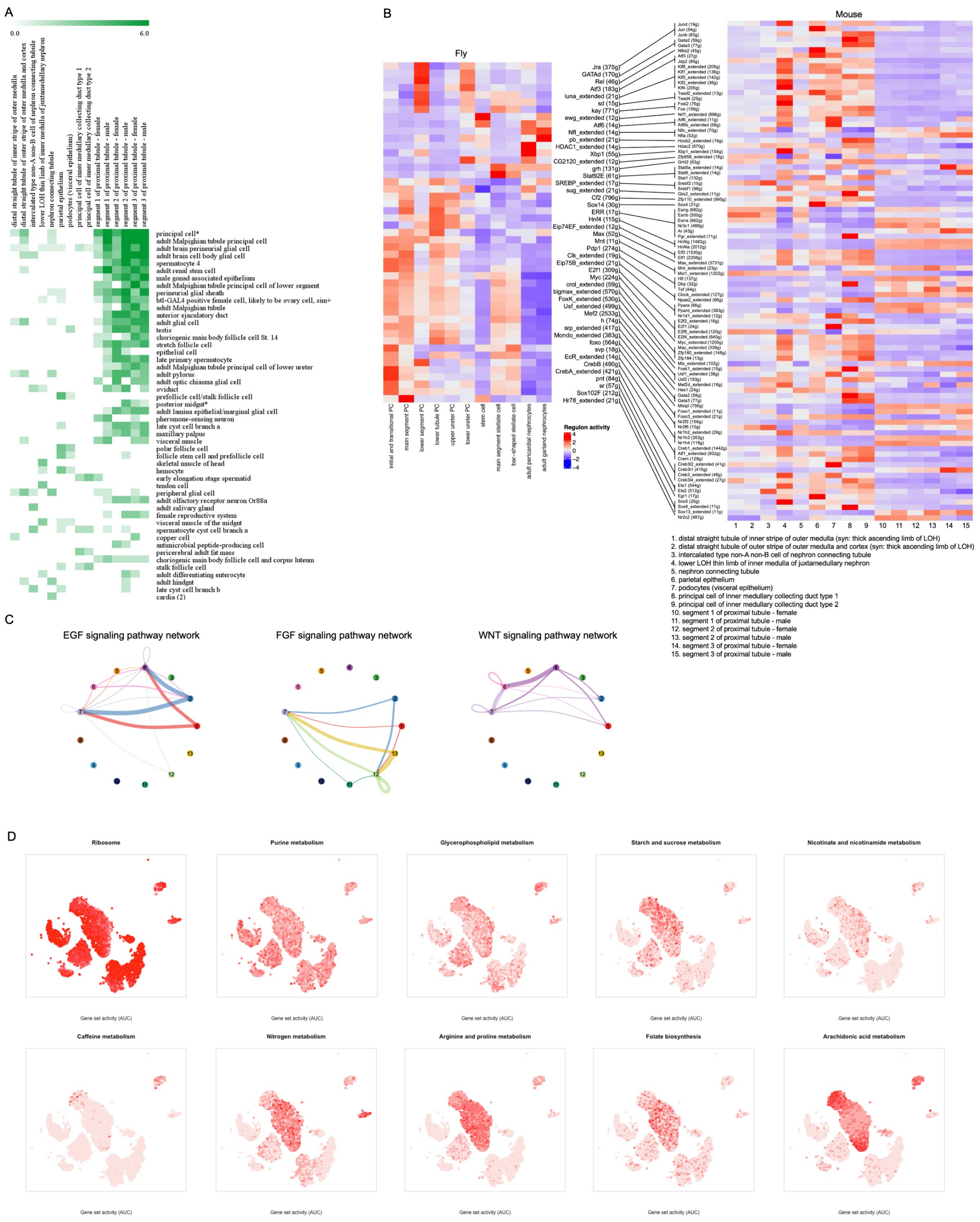

Figure S9. Comparison of cell clusters, transcription factors, cell-cell communication, and metabolic pathways between mouse and fly kidney. (A) Comparison of mouse kidney cells (15 cell clusters) with cells from the entire FCA (252 cell clusters) based on the top 50 markers of each cell cluster. (B) Comparison of the TFs from the SCENIC analysis: 47 fly genes and their mouse orthologs. (C) Three representative circle plots of cell-cell communication events between mouse kidney cell clusters. (D) The upper panel is for highly/ubiquitously expressed metabolic pathways in the mouse kidney, which were also highly expressed in the fly kidney. Lower panel is for five specifically expressed pathways in 1-2 cell clusters in the mouse kidney that are consistent with the fly data.

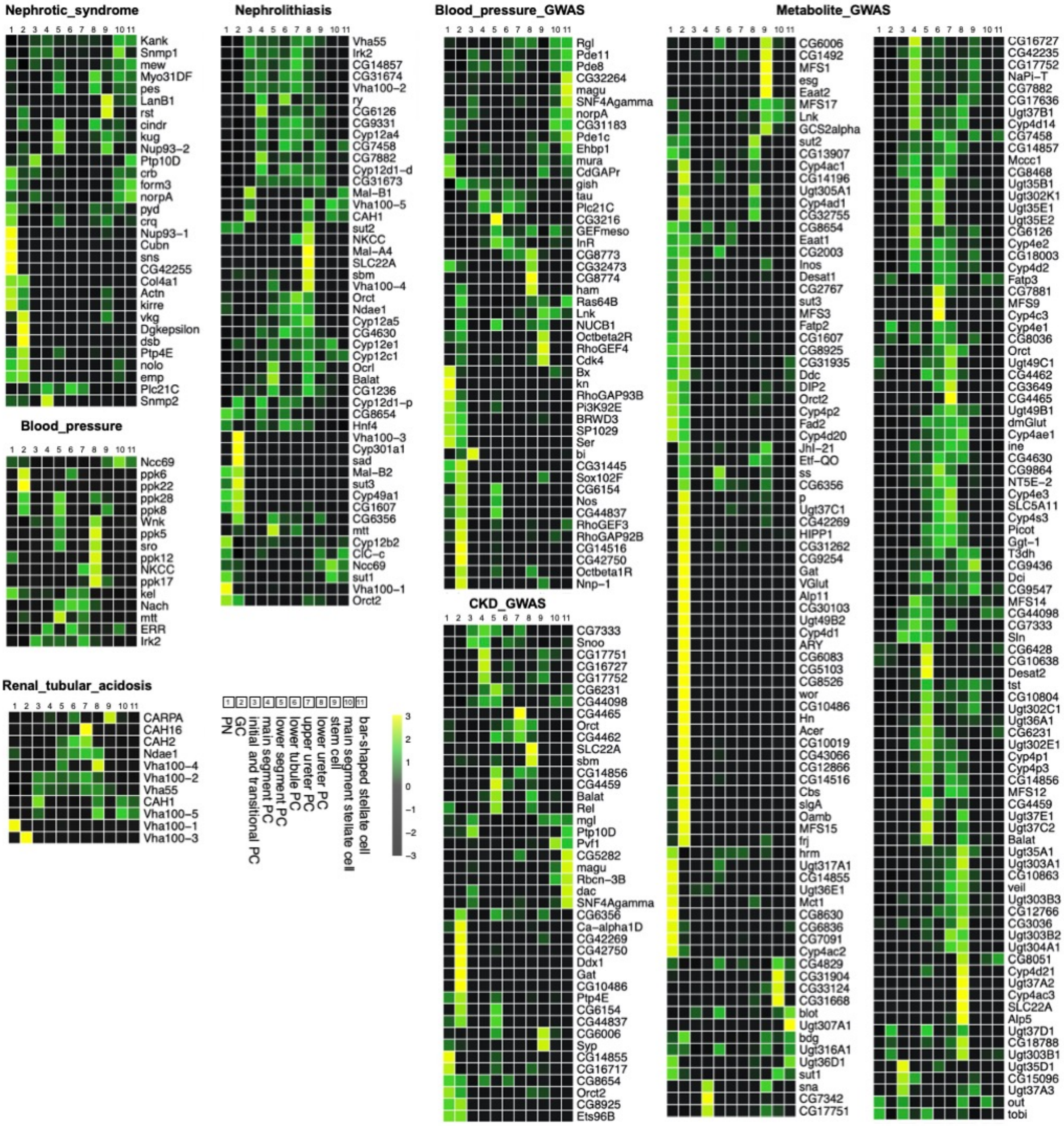

Figure S10. Expression of fly orthologs of human kidney disease-associated genes in specific fly kidney cell types. Average expression in single cell clusters of fly orthologs of human monogenic disease genes and complex-trait genes identified from genome-wide association studies (GWAS) (39). Mean expression values of the genes were calculated for each cluster. The color scheme is based on z-score distribution (-3 < z-scores < 3). Each row in the heat map represents one gene and each column a single cell type.

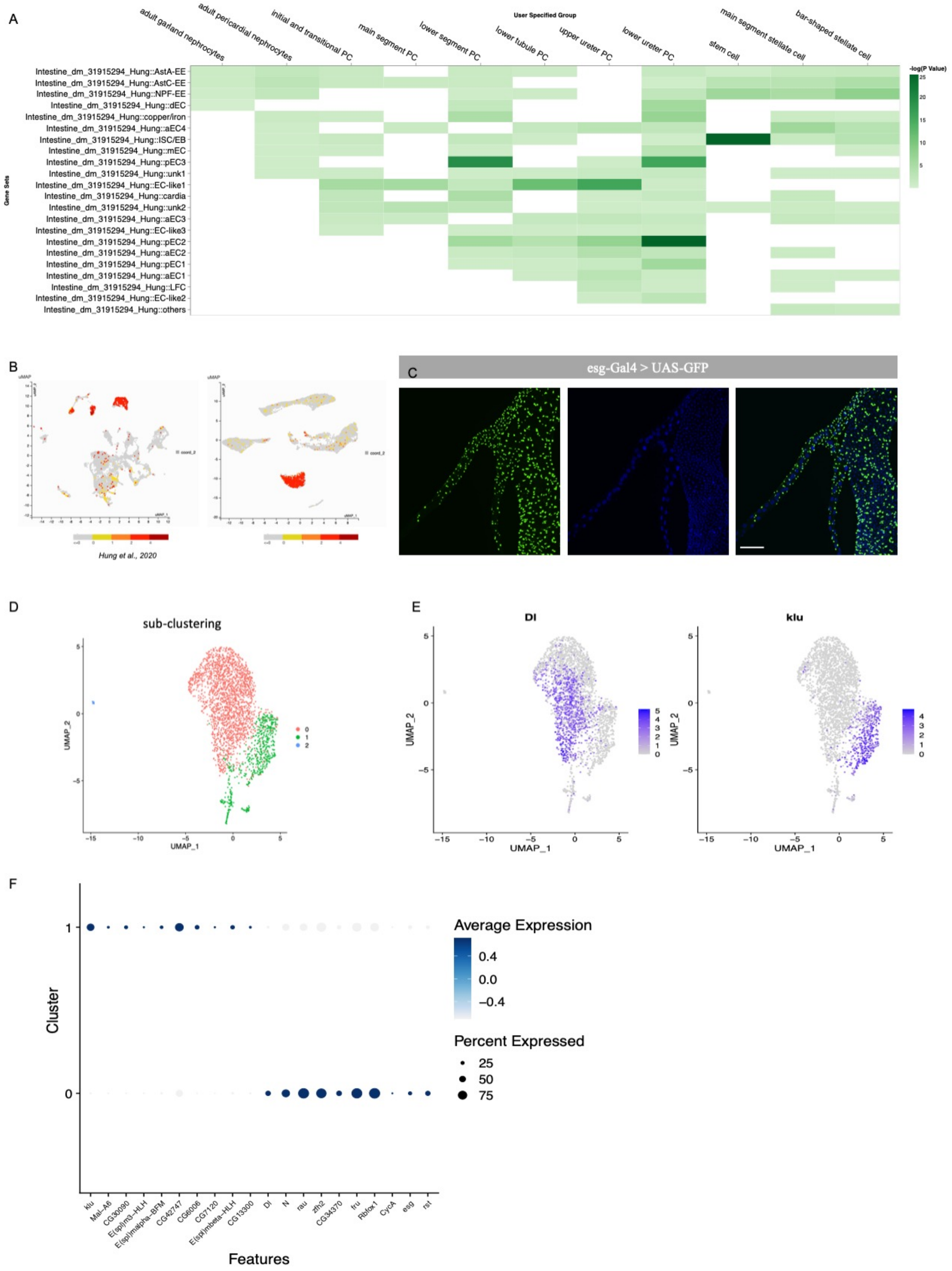

Figure S11. Comparison of renal stem cell and intestinal stem cell clusters. (A) snRNA-seq midgut clusters are from (25). The X axis shows the 11 integrated clusters from Fig. 1B. The Y axis shows the midgut clusters from (25). Colors represent gene expression similarities. Note that RSCs are highly similar to ISCs. In addition, lower ureter PCs share high similarity with pEC2 (posterior enterocytes). (B) *escargot (esg)* expression in the gut and Malpighian tubule UMAPs. (C) *esg* expression in the

Malpighian tubules visualized using esg-Gal4 driving UAS-GFP expression. Scale bars = 100 μm. (D) UMAP distribution of different sub-clusters of RSCs. (E) *Delta (Dl)* and *klumpfuss (klu)* expression in the RSC UMAP subclusters. (F) Dot plot showing the expression levels and percentage of cells expressing the various markers in the *Dl* and *klu* sub-clusters.

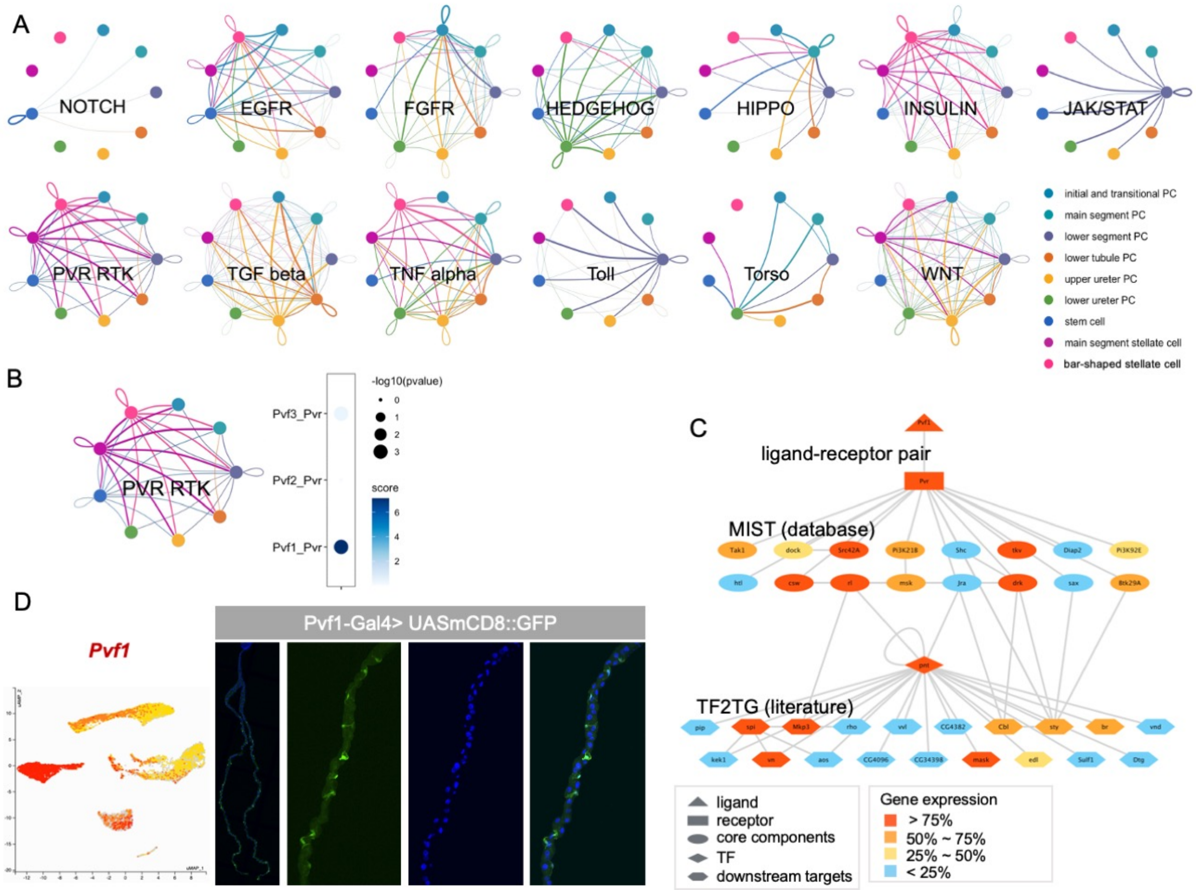

Figure S12. Cell–cell communication analysis in the adult fly kidney. (A) Network of 13 signaling pathways in fly kidney cell clusters. Each of the 11 cell clusters is displayed in a different color. The predicted interaction between two clusters is indicated by a color curve. The thickness of the curve indicates the strength of the interaction. The full list of predicted ligand/receptor pair genes can be found in Dataset S10. (B) Ligand-receptor interaction between *Pvf1* and its receptor *Pvr* in main segment SCs and main segment PCs. The panel on the right shows a dot plot of the interaction score and specificity of ligand-receptor pairs between main segment principal cell cluster and main segment stellate cell cluster. (C) *Pvr-Pvf1* interaction network based on MIST and TF2TG. (D) *Pvf1* expression in SCs visualized using Pvf1-Gal4 driving UAS-mCD8::GFP expression.

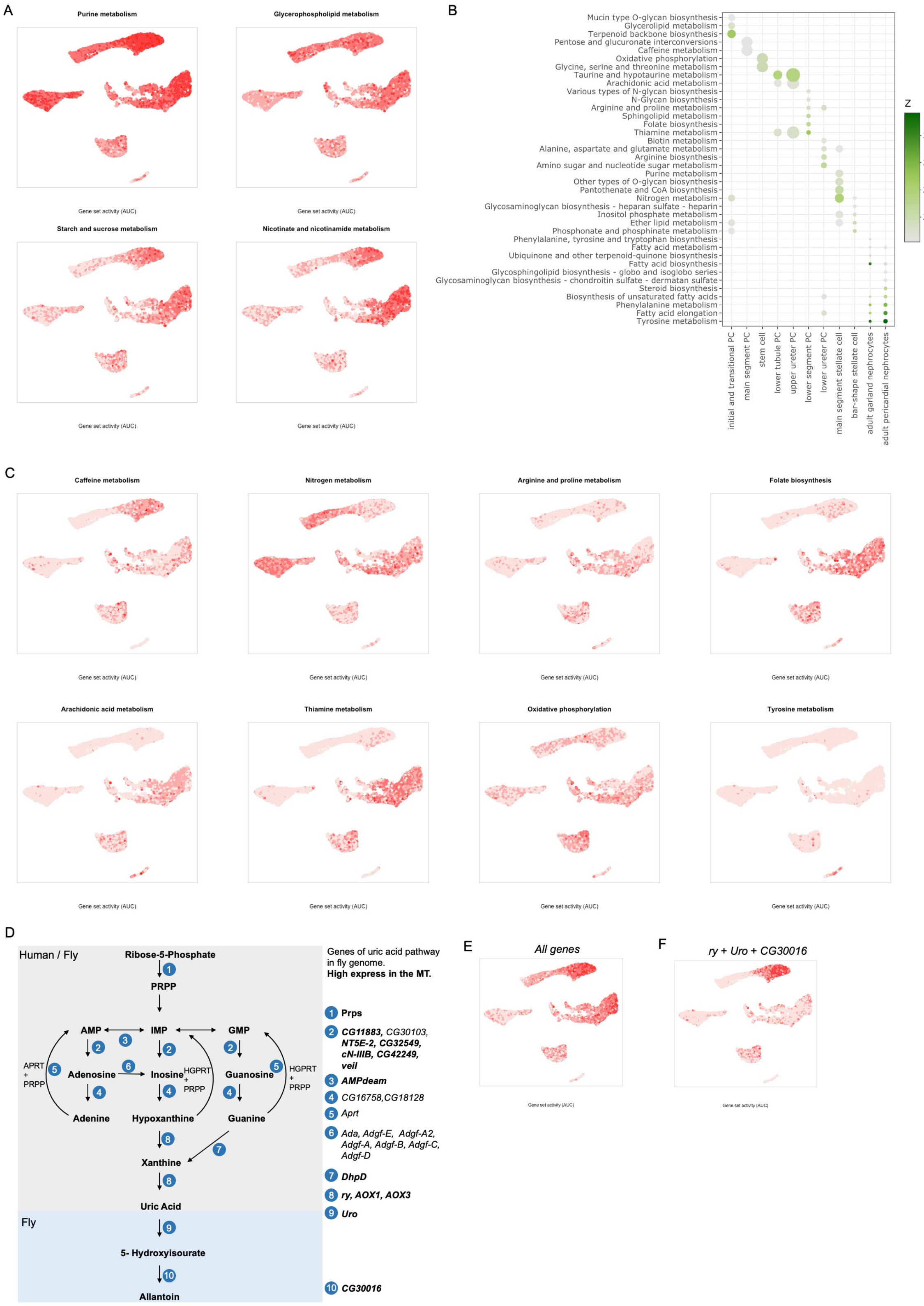

Figure S13. Metabolic pathways analysis. (A) Four representatives of highly expressed metabolic pathways in the fly kidney. (B) The statistical analysis identified 38 metabolic pathways specifically expressed in 1-2 cell types. (C) Eight representatives of specifically expressed pathways in the fly kidney. Color intensity reflects pathway activity. (D) Gene distribution of the Uric Acid pathway in the fly kidney.

The end product is uric acid in human and allantoin in the fly. Right panel: the enzymes involved at each step. (E) The UMAP plot shows the gene set activity of all genes in the uric acid pathway. (F) Gene set activity of the last three steps, *rosy* (*ry*), *Urate oxidase* (*Uro*) and *CG30016,* visualized by UMAP plots.

SI references

1. H. Li *et al*., Fly Cell Atlas: a single-cell transcriptomic atlas of the adult fruit fly. *bioRxiv.* https://doi.org/10.1101/2021.07.04.451050 (2021).
2. S. Aibar *et al*., SCENIC: single-cell regulatory network inference and clustering. Nat. Methods *14*, 1083–1086 (2017).
3. Y.F. Liu *et al*., FlyPhoneDB: An integrated web-based resource for cell-cell communication prediction in *Drosophila*. *Genetics* iyab235 (2021).
4. Y. Hu *et al*., Molecular Interaction Search Tool (MIST): an integrated resource for mining gene and protein interaction data. *Nucleic. Acids Res.* **46**, D567–D574 (2018).
5. D. Otasek, J.H. Morris, J. Bouças, A.R. Pico, B. Demchak, Cytoscape Automation: empowering workflow-based network analysis. *Genome Biol.* **20**, 185 (2019).
6. S. Jin *et al*., Inference and analysis of cell-cell communication using CellChat. *Nat. Commun.* **12**, 1088 (2021).
7. A.J. Tarashansky *et al*., Mapping single-cell atlases throughout Metazoa unravels cell type evolution. *Elife* **10**, e66747 (2021).
8. Y. Hu *et al*., DRscDB: A single-cell RNA-seq resource for data mining and data comparison across species. *Comput. Struct. Biotechnol. J.* **19**, 2018–2026 (2021).
9. W. Song, *et al*., Tumor-derived ligands trigger host wasting via MEK activation. *Dev. Cell* **48**, 277–286 (2019).
10. Y. Kwon, *et al*., Systemic organ wasting induced by localized expression of the secreted insulin/IGF antagonist ImpL2. *Dev. Cell* **33**, 36–46 (2015).
11. S.A. Davies *et al*., Analysis and inactivation of vha55, the gene encoding the vacuolar ATPase B-subunit in *Drosophila melanogaster* reveals a larval lethal phenotype. *J. Biol. Chem.* **271**, 30677–30684 (1996).
12. J.P. Day *et al*., Identification of two partners from the bacterial Kef exchanger family for the apical plasma membrane V-ATPase of Metazoa. *J. Cell Sci.* **121**, 2612–2619 (2008).
13. M.J. O'Donnell, S.H. Maddrell, Fluid reabsorption and ion transport by the lower Malpighian tubules of adult female *Drosophila*. *J. Exp Biol.* **198**, 1647–1653 (1995).
14. L.S. Torrie *et al*., Resolution of the insect ouabain paradox. *Proc. Natl. Acad. Sci. U.S.A.***101**, 13689–13693 (2004).
15. J.M. Evans, A.K. Allan, S.A. Davies, J.A.T. Dow, Sulphonylurea sensitivity and enriched expression implicate inward rectifier K+ channels in Drosophila melanogaster renal function. *J. Exp Biol.* **208**, 3771–3783 (2005).
16. Y. Wu, M. Baum, C.L. Huang, A.R Rodan, Two inwardly rectifying potassium channels, Irk1 and Irk2, play redundant roles in Drosophila renal tubule function. *Am. J. Physiol. Regul. Integr. Comp. Physiol.* **309**, R747–756 (2015).
17. D.R. Swale *et al*., An insecticide resistance-breaking mosquitocide targeting inward rectifier potassium channels in vectors of Zika virus and malaria. *Sci. Rep.* **6**, 36954 (2016).

18. C.M. Sciortino, L.D. Shrode, B.R. Fletcher, P.J. Harte, M.F. Romero, Localization of endogenous and recombinant Na (+)-driven anion exchanger protein NDAE1 from *Drosophila melanogaster*. *Am. J. Physiol. Cell Physiol.* **281**, C449–463 (2001).
19. S.M. Linton, M.J. O'Donnell, Contributions of K+: Cl- cotransport and Na+/K+-ATPase to basolateral ion transport in malpighian tubules of *Drosophila melanogaster*. *J. Exp. Biol.* **202**, 1561–1570 (1999).
20. A.R. Rodan, M. Baum, C.L. Huang, The *Drosophila* NKCC Ncc69 is required for normal renal tubule function. *Am. J. Physiol. Cell Physiol.* **303**, C883–894 (2012).
21. C. Wang, A.C. Spradling, An abundant quiescent stem cell population in *Drosophila* Malpighian tubules protects principal cells from kidney stones. *Elife* **9**, e54096 (2020).
22. S.R. Singh, W. Liu, S.X. Hou, The adult *Drosophila* malpighian tubules are maintained by multipotent stem cells. *Cell Stem Cell* **1**, 191–203 (2007).
23. S. Takashima, M. Paul, P. Aghajanian, A. Younossi-Hartenstein, V. Hartenstein, Migration of *Drosophila* intestinal stem cells across organ boundaries. *Development* **140**, 1903–1911 (2013).
24. K. Xu, X. Liu, Y. Wang, C. Wong, Y. Song, Temporospatial induction of homeodomain gene cut dictates natural lineage reprogramming. *Elife* **7**, e33934 (2018).
25. R.J. Hung *et al*., A cell atlas of the adult *Drosophila* midgut. *Proc. Natl. Acad. Sci. U.S.A.* **117**,1514-1523 (2020).
26. M.A. Loza-Coll, T.D. Southall, S.L. Sandall, A.H. Brand, D.L. Jones, Regulation of *Drosophila* intestinal stem cell maintenance and differentiation by the transcription factor Escargot. *EMBO J.* **33**, 2983–2996 (2014).
27. B. Ohlstein, A. Spradling, The adult *Drosophila* posterior midgut is maintained by pluripotent stem cells. *Nature* **439**, 470–474 (2006).
28. C.A. Micchelli, N. Perrimon, Evidence that stem cells reside in the adult *Drosophila* midgut epithelium. *Nature* **439**, 475–479 (2006).
29. L. Couturier, K. Mazouni, F. Corson, F. Schweisguth, Regulation of Notch output dynamics via specific E(spl)-HLH factors during bristle patterning in *Drosophila*. *Nat. Commun.* **10**, 3486 (2019).
30. Y. Lu, Z. Li, Notch signaling downstream target E(spl)mbeta is dispensable for adult midgut homeostasis in *Drosophila*. *Gene* **560**, 89–95 (2015).
31. Z. Li, S. Liu, Y. Cai. Differential Notch activity is required for homeostasis of malpighian tubules in adult *Drosophila*. *J. Genet. Genomics* **41**, 649–652 (2014).
32. Z. Li, S. Liu, Y. Cai, EGFR/MAPK signaling regulates the proliferation of Drosophila renal and nephric stem cells. *J. Genet. Genomics* **42**, 9–20 (2015).
33. C.E. Dent, G.R. Philpot, Xanthinuria, an inborn error (or deviation) of metabolism. *Lancet* **266**, 182–185 (1954).
34. K. Ichida *et al*., Identification of two mutations in human xanthine dehydrogenase gene responsible for classical type I xanthinuria. *J. Clin. Invest.* **99**, 2391–2397 (1997).
35. J. Miller *et al*., *Drosophila melanogaster* as an emerging translational model of human nephrolithiasis. *J. Urol.* **190**,1648–1656 (2013).
36. A.K. Mandal, D.B. Mount, The molecular physiology of uric acid homeostasis. *Annu. Rev. Physiol.* **77**, 323–345 (2015).
37. H.K. Mitchell, E. Glassman, Hypoxanthine in rosy and maroon-like mutants of *Drosophila melanogaster*. *Science* **129**, 268 (1959).
38. Y.H. Hobani, Metabolomic analyses of *Drosophila* models for human renal disease. *PhD theses* http://theses.gla.ac.uk/id/eprint/3222 (2012).

39. J. Park *et al.*, Single-cell transcriptomics of the mouse kidney reveals potential cellular targets of kidney disease. *Science* **360**, 758–763 (2018).

Legends for Datasets S1 to S10

Dataset S1. Basic QC and sequencing information.
Dataset S2. Differentially expressed genes in each cluster. Only positive marker genes are shown.
Dataset S3. Table of validated markers, from previous studies and this study, allowing assignment of clusters to cell types or regions.
Dataset S4. Full list of regulons and their respective predicted target genes.
Dataset S5. List of cell type-specific transcription factors.
Dataset S6. Gene pairs for fly and mouse cell type mappings.
Dataset S7. Gene pairs for fly and planarian cell type mappings.
Dataset S8. GO terms of the six PC cell clusters.
Dataset S9. List of gene pairs for cell-cell communication predictions.
Dataset S10. Gene list comparison of RSCs and ISCs.
